# GLI1 activates pro-fibrotic pathways in myelofibrosis fibrocytes

**DOI:** 10.1038/s41419-022-04932-4

**Published:** 2022-05-20

**Authors:** Taghi Manshouri, Ivo Veletic, Ping Li, C. Cameron Yin, Sean M. Post, Srdan Verstovsek, Zeev Estrov

**Affiliations:** 1 Department of Leukemia, The University of Texas MD Anderson Cancer Center, Houston, Texas USA; 2 Department of Hematopathology, The University of Texas MD Anderson Cancer Center, Houston, Texas USA

**Keywords:** Myeloproliferative disease, Growth factor signalling, Apoptosis, Monocytes and macrophages, Mechanisms of disease

## Abstract

Bone marrow (BM) fibrosis was thought to be induced exclusively by mesenchymal stromal cells (MSCs). However, we and others found that neoplastic fibrocytes induce BM fibrosis in myelofibrosis (MF). Because glioma-associated oncogene-1 (GLI1), an effector of the Hedgehog pathway, plays a role in the induction of BM fibrosis, we wondered whether GLI1 affects fibrocyte-induced BM fibrosis in MF. Multiplexed fluorescence immunohistochemistry analysis of MF patients’ BM detected high levels of GLI1 in MF fibrocytes compared to MSCs or normal fibrocytes. Immunostaining, RNA in situ hybridization, gene expression analysis, and western immunoblotting detected high levels of GLI1 and GLI1-induced matrix metalloproteases (MMP) 2 and 9 in MF patients BM-derived cultured fibrocytes. Similarly, MF patients’ BM-derived GLI1^+^ fibrocytes were found in BMs and spleens of MF xenograft mice. GLI1 silencing reduced the levels of MMP2/9, phosphorylated SMAD2/3, and procollagen-I, and knockdown or inhibition of GLI1 decreased fibrocyte formation and induced apoptosis of both fibrocytes and fibrocyte progenitors. Because Janus kinase (JAK)2-induced STAT3 is constitutively activated in MF and because STAT3 induces GLI1 expression, we sought to determine whether STAT3 activates GLI1 in MF fibrocytes. Imaging analysis detected phosphotyrosine STAT3 in MF patients’ BM fibrocytes, and transfection of fibrocytes with STAT3-siRNA or treatment with a JAK1/2 inhibitor ruxolitinib reduced GLI1 and MMP2/9 levels. Chromatin immunoprecipitation and a luciferase assay revealed that STAT3 induced the expression of the GLI1 gene in both MF BM fibrocytes and fibrocyte progenitors. Together, our data suggest that STAT3-activated GLI1 contributes to the induction of BM fibrosis in MF.

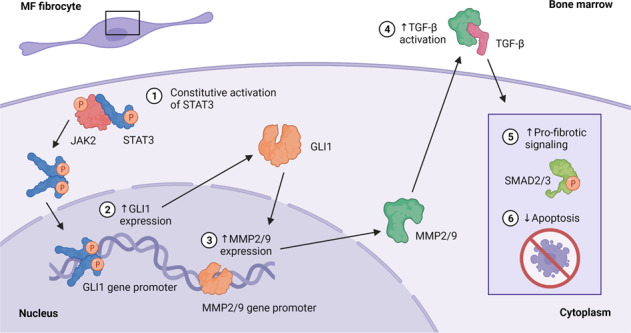

## Introduction

Primary myelofibrosis (PMF), polycythemia vera (PV), and essential thrombocytosis (ET) are clonal myeloproliferative neoplasms (MPNs) [1]. Like PMF, post-PV, and post-ET myelofibrosis (MF) is characterized by progressive bone marrow (BM) fibrosis [2, 3]. Contrary to the current consensus, according to which BM fibrosis in MF is induced exclusively by mesenchymal stromal cells (MSCs) [3–5], we and other investigators found that neoplastic monocyte-derived fibrocytes induce BM fibrosis in MF [6–8]. We detected large numbers of neoplastic monocyte-derived collagen- and fibronectin-producing fibrocytes in the BM of patients with MF and showed that engrafted MF patients’ neoplastic monocyte-derived fibrocytes induced BM fibrosis in xenotransplant mice [6]. Similarly, Ozono et al. showed that the vast majority of cells in the BM and spleen of mice with mutated Janus kinase (JAK)-2 (*Jak2*^V617F^)-induced MF were fibrocytes [8]. However, to what degree neoplastic fibrocytes, as opposed to non-neoplastic MSCs, contribute to the induction of BM fibrosis in MF is currently unknown.

Glioma-associated oncogene-1 (GLI1), a downstream effector of the Hedgehog (Hh) signaling pathway, commonly expressed in embryonic cells [9, 10], is also expressed in MF fibrocytes [6]. Cells expressing GLI1 were detected in the BM of thrombopoietin- and JAK2-induced murine models of BM fibrosis and ablation of GLI1^+^ cells abolished BM fibrosis in these animals’ tissues [11, 12]. Similarly, GLI1 levels in MF patients’ BM cells correlated with the degree of BM fibrosis [11]. However, whether GLI1 plays a role in fibrocyte-induced BM fibrosis is unknown.

In MF the JAK-signal transducer and activator of transcription (STAT) pathway is constitutively activated [13, 14]. We recently found that STAT3 binds the GLI1 gene promoter and induces the expression of GLI1 [15]. Therefore, we sought to determine whether STAT3 induces the expression of GLI1 in neoplastic MF fibrocytes and whether STAT3-activated GLI1 plays a role in the induction of BM fibrosis.

## Results

### MF fibrocytes, but not MSCs, express high levels of GLI1

**Fig. 1:**
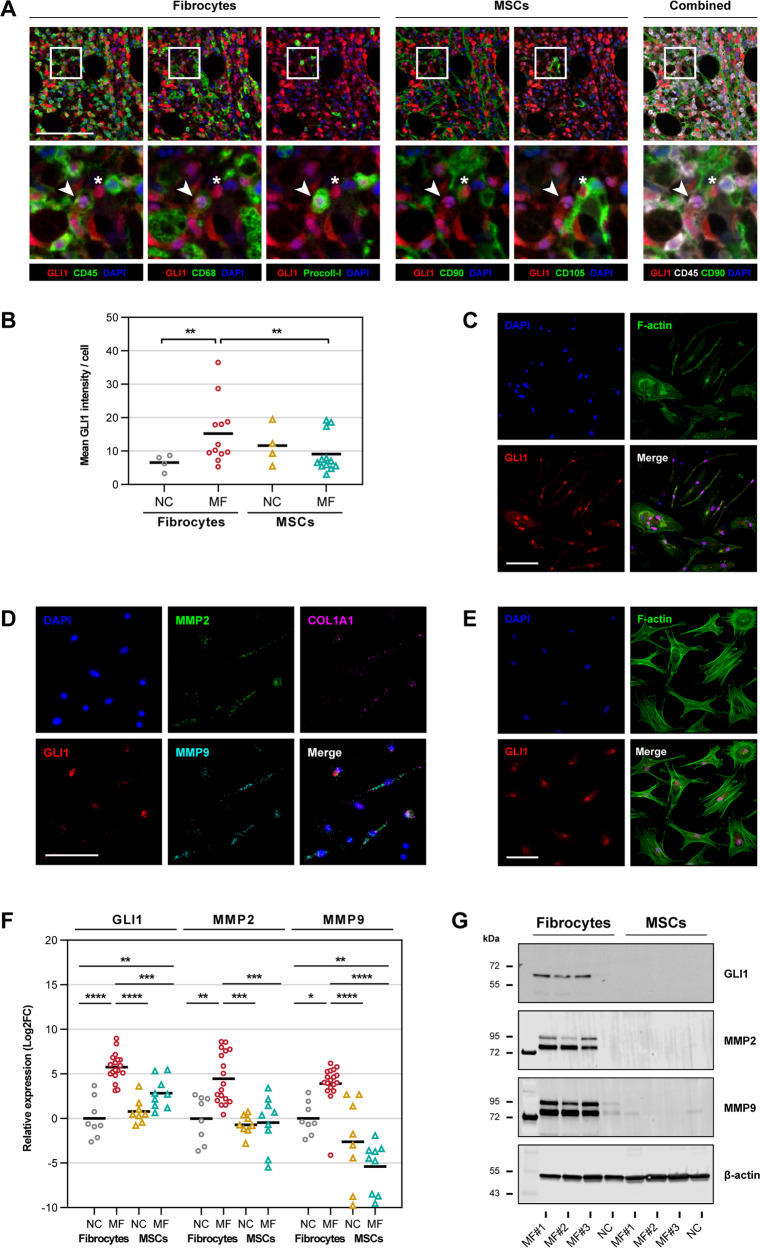
GLI1 levels in BM fibrocytes and MSCs of MF patients in situ and in vitro. **A** Representative micrographs of fibrocytes (left panel) and MSCs (right panel) in BM biopsy sections of an MF patient were analyzed using mfIHC. In addition to GLI1 (Opal 520, pseudo-colored red), fibrocytes were stained using CD45 (Opal 690), CD68 (Opal 620), and procollagen-I (Opal 570) antibodies and MSCs were stained using CD90 (Opal 650) and CD105 (Opal 540) antibodies (pseudo-colored green). Shown in zoomed-in panels are a representative fibrocyte (arrows) and an MSC (asterisks). Fibrocytes are typified by a granular staining pattern inside the BM cavity, whereas MSCs by a linear staining pattern within the endosteal or perivascular space. GLI1 co-localized with the cell nuclei (DAPI, pseudo-colored blue) in both fibrocytes and MSCs. The right-most panel depicts combined CD45 (hematopoietic) and CD90 (MSC) markers (pseudo-colored white and green, respectively) together with GLI1 (pseudo-colored red). **B** Quantitative analysis of GLI1 signal intensities in BM biopsy sections of MF patients (*n* = 7) and NCs (*n* = 4) by mfIHC. Whole BM tissue sections were imaged at ×400 resolution and individual fibrocytes (CD45^+^/CD68^+^/procollagen-I^+^) and MSCs (CD90^+^/CD105^+^) were detected using an unsupervised tissue and cell segmentation algorithm. GLI1 signal intensity was quantitated in each cell and mean signal intensity of all detected fibrocytes and MSCs was calculated for each BM biopsy. Horizontal lines denote the mean. The statistical significance of the difference between groups was calculated using the one-way ANOVA followed by Tukey’s post hoc tests. **C** Representative micrographs of cultured fibrocytes from an MF patient were analyzed using immunofluorescence. BM LDCs aspirated from 11 MF patients were cultured in conditions favoring differentiation to fibrocytes and co-stained for GLI1 (Alexa Fluor 594, pseudo-colored red) and F-actin (Alexa Fluor 488, pseudo-colored green). Like in MF BM biopsies GLI1 is highly expressed in cultured MF fibrocytes and co-localizes with the cells’ nuclei (DAPI, pseudo-colored blue). **D** Representative micrographs of cultured fibrocytes of an MF patient were analyzed using multiplexed fluorescence RNA in situ hybridization. BM LDCs from three MF patients were cultured in the fibrocyte-forming assay and hybridized using GLI1 (Opal 520, pseudo-colored red), MMP2 (Opal 620, pseudo-colored green), MMP9 (Opal 570, pseudo-colored cyan), and procollagen-I (*COL1A1*; Opal 690, pseudo-colored magenta) probes. mRNA of all 4 analyzed genes was detected in MF fibrocytes. DAPI (pseudo-colored blue) was the nuclear counterstain. **E** Representative micrographs of cultured MSCs of an MF patient were analyzed using fluorescence immunostaining. BM LDCs aspirated from five MF patients were cultured in conditions favoring differentiation to MSCs and co-stained for GLI1 (Alexa Fluor 594, pseudo-colored red) and F-actin (Alexa Fluor 488, pseudo-colored green). DAPI (pseudo-colored blue) was the nuclear counterstain. **F** Quantitation of mRNA expression of GLI1 and the GLI1-target genes MMP2 and MMP9 in cultured fibrocytes or MSCs of MF patients and NCs as analyzed by qRT-PCR. mRNA levels in MF fibrocytes (*n* = 18) were compared to MF MSCs (*n* = 9) and NC fibrocytes and MSCs (*n* = 8 each). Horizontal lines denote mean. The statistical significance of differences between groups was calculated by one-way ANOVA followed by Tukey’s post hoc tests. **G** Protein levels of GLI1, MMP2, and MMP9 in cultured fibrocytes and MSCs of MF patients (*n* = 3) and an NC were assessed by using western immunoblotting. GLI1, MMP2, and MMP9 proteins were detected only in MF fibrocytes. All target proteins were detected by sequential probing of the same blot and β-actin was used as a loading control. All micrographs were adjusted for brightness and contrast linearly and equally between groups. Scale bars represent 100 µm. *P < 0.05; **P < 0.01; ***P < 0.001; ****P < 0.0001, respectively.

A recent study suggested that GLI1 enhances MSC-induced BM fibrosis [11, 12]. Because we and other investigators found that fibrocytes, rather than MSCs, induce BM fibrosis [6–8] we sought to assess the levels of GLI1 in MF patients’ fibrocytes and MSCs. To do this, we obtained BM biopsy specimens from seven patients with MF and four normal controls (NCs) and studied them using multiplexed fluorescence immunohistochemistry (mfIHC). MSCs are stellate-shaped cells localized in proximity to arterioles and sinusoids or adjacent to bone [16], whereas fibrocytes are spindle-shaped cells usually found within the BM cavity [17]. Consistent with previously reported data [11], we detected GLI1 in CD90^+^/CD105^+^ MSCs [16] in perivascular and endosteal spaces of MF patients’ BM tissue (Fig. 1A, right panel) and in CD45^+^/CD68^+^/procollagen-I^+^ fibrocytes [18] located within the BM cavity (Fig. 1A, left panel). Then, by using quantitative imaging analysis we found that GLI1 signal intensity levels were 3.1-fold higher in BM fibrocytes than in MSCs and that MF patients’ fibrocytes had significantly higher GLI1 signal intensity levels compared to BM fibrocytes of healthy individuals (*P* < 0.01 in both). Conversely, GLI1 intensity levels in BM MSCs of MF patients were similar to those of BM MSCs of healthy individuals (Fig. 1B).

To delineate these findings, we incubated BM low-density cells (LDCs) of 39 MF patients in culture conditions that induce differentiation to either fibrocytes or MSCs. By using fluorescence immunostaining we detected GLI1 nuclear signals in both cell types (Fig. 1C, E) and by using multiplexed fluorescence RNA in situ hybridization we found that MF fibrocytes expressed mRNA of GLI1, the GLI1-target genes matrix metalloproteases (MMPs) 2 and 9, and of procollagen-I (*COL1A1*) (Fig. 1D). To confirm these findings and to assess mRNA and protein levels of GLI1 and its target genes, we used quantitative real-time (qRT)-PCR and western immunoblotting. Consistent with our previous data, we found high mRNA (Fig. 1F) and protein (Fig. 1G) levels of GLI1, MMP2, and MMP9 in MF fibrocytes. GLI1 mRNA was detected in MF MSCs but unlike MF fibrocytes, and similar to MSCs of NCs, MMP2, and MMP9 mRNA levels were significantly decreased (Fig. 1F). Furthermore, neither GLI1 nor MMP2 or MMP9 proteins were detected in MF MSCs (Fig. 1G). Taken together, our data suggest that MF patients’ BM cells that express GLI1 and GLI1-target genes are neoplastic fibrocytes rather than MSCs.

### MF patients’ BM LDCs engrafted in NSG mice give rise to GLI1^+^ fibrocytes

**Fig. 2:**
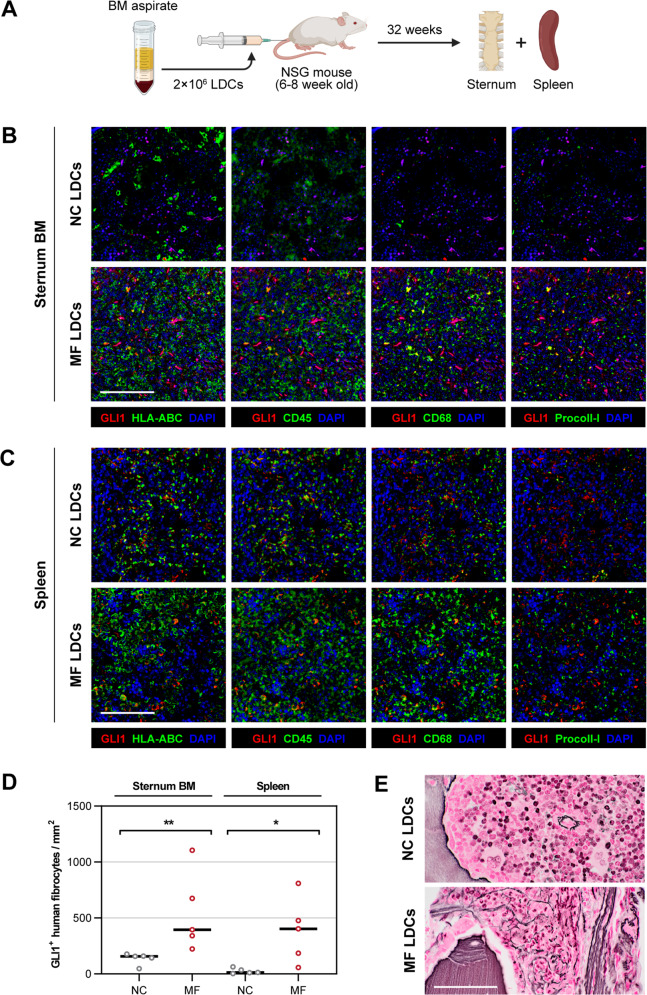
Human MF BM cells engraft in NSG mice and give rise to GLI1^+^ fibrocytes in vivo. **A** Diagram of a MF xenograft mouse model was generated to study GLI1 expression in MF fibrocytes. BM LDCs from MF patients (*n* = 2) or NCs (*n* = 2) were transplanted into NSG mice (*n* = 5 per group) at 6–8 weeks of age. Following successful engraftment assessed by flow cytometry, sternum and spleen were harvested, and tissue sections were analyzed using mfIHC. **B**, **C** Representative micrographs of GLI1^+^ human fibrocytes in the BM (**B**) and spleen (**C**) of MF xenograft mice. In addition to GLI1 (Opal 480, pseudo-colored red), tissues were co-stained using HLA-ABC (Opal 780), CD45 (Opal 570), CD68 (Opal 520), and procollagen-I (Opal 690) antibodies (pseudo-colored green). Shown are composites of each phenotype marker along with GLI1 for reference. Large clusters of GLI1^+^ human fibrocytes, defined as HLA-ABC^+^/CD45^+^/CD68^+^/procollagen-I^+^ cells, were found in the BM and spleen tissue of NSG mice transplanted with MF BM LDCs, but only scattered GLI1^+^ fibrocytes were detected in the NC-transplanted mice. DAPI (pseudo-colored blue) was used as a nuclear counterstain. **D** Quantitation of GLI1^+^ human fibrocytes in the BM and spleen of xenograft mice (*n* = 5 per group) engrafted with human MF or NC BM LDCs. Whole tissue sections were imaged at ×400 resolution, deconvoluted, and analyzed using an unsupervised segmentation and phenotyping algorithm. Numbers of GLI1^+^/HLA-ABC^+^/CD45^+^/CD68^+^/procollagen-I^+^ cells were calculated per unit area using unbiased counting frames. Horizontal lines denote median. The significance of the difference between MF- and NC-xenografts was assessed using the Mann–Whitney test. **E** Representative micrographs of reticulin fibrosis in the BM tissue section of the xenograft mice analyzed using Gomori’s silver stain method. Bundles of reticulin fibers were detected in the BM of mice transplanted with BM LDCs from MF patients but not in mice transplanted with NC BM cells. Nuclear fast red was used as a counterstain. All micrographs were adjusted for brightness and contrast linearly and equally between groups. Scale bars represent 100 µm. *P < 0.05; **P < 0.01, respectively.

Using a xenograft mouse model, we previously demonstrated that MF patients’ BM LDCs engraft in immunodeficient NOD-*scid* gamma (NSG) mice and that neoplastic monocyte-derived human fibrocytes induce BM fibrosis in the engrafted animals [6, 19]. Since the JAK-STAT pathway is constitutively activated in MF and because we recently found that STAT3 induces the expression of GLI1 [13–15], we sought to determine whether MF patients’ BM cells give rise to GLI1^+^ fibrocytes that induce BM fibrosis in vivo. To answer this question, we engrafted NSG mice with human BM LDCs and harvested from the xenograft mice sternums and spleens (Fig. 2A). After staining tissue sections using mfIHC, we detected large clusters of GLI1-positive HLA-ABC^+^/CD45^+^/CD68^+^/procollagen-I^+^ cells, corresponding to human fibrocytes, in the animals’ BM and spleen (Fig. 2B, C). Quantitative analysis of whole tissue sections showed significantly high number of GLI1^+^ human fibrocytes in both sternum BM and spleen sections of mice transplanted with MF patients’ BM LDCs (*P* < 0.05 and *P* < 0.01, respectively; Fig. 2D). In addition, analysis of BM tissue sections of the xenograft mice using Gomori’s silver stain showed significant reticulin fibrosis in the BM of the NSG mice engrafted with MF LDCs (Fig. 2E). Taken together, our data suggest that monocytes within the transplanted MF patients’ BM LDCs differentiate into GLI1^+^ fibrocytes that likely induce BM fibrosis in the xenograft mice.

### Inhibition of GLI hampers fibrocyte formation

**Fig. 3:**
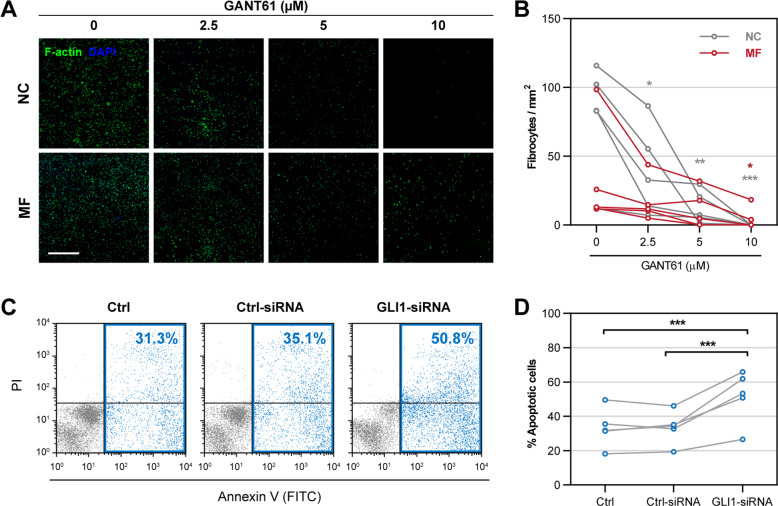
GLI1 is required for fibrocyte formation in vitro. **A** Representative micrographs of fibrocytes formed after exposure of BM LDCs to 0, 2.5, 5, and 10 μM GANT61 and analyzed by fluorescence cytochemistry. BM LDCs from MF patients and NCs were cultured in conditions favoring differentiation to fibrocytes and GANT61 was added 24 h following culture initiation. After 7 days cells were stained for F-actin (Alexa Fluor 488, pseudo-colored green) and their nuclei were counterstained using DAPI (pseudo-colored blue). As shown, 7-day treatment with GANT61 reduced the numbers of both MF and NC fibrocytes in a dose-dependent manner. All micrographs were adjusted for brightness and contrast linearly and equally between groups. The scale bar represents 1 mm. **B** Quantitation of fibrocyte formation following 7-day treatment with GANT61. Fibrocyte numbers were assessed by using an unsupervised cell segmentation algorithm and the mean cell number per unit area was calculated for each well. The significance of change at different concentrations of GANT61 was assessed using repeated-measures ANOVA followed by Dunnett’s multiple comparisons test. **C** Representative annexin V/PI flow cytometry plots of GLI1-silenced BM LDCs from an MF patient. BM LDCs from MF patients were transfected with GLI1-siRNA or Ctrl-siRNA for 24 h and stained using annexin V (FITC) and PI. Untreated BM LDCs from the same patient served as a negative control (Ctrl). To measure the percentage of apoptotic cells annexin V-positive cells were gated in each sample. As shown, the fraction of apoptotic cells was 50% higher in GLI1-silenced LDCs than in both controls. **D** Quantitation of apoptotic cell fractions after GLI1 silencing of LDCs from MF patients (*n* = 5). The difference between siRNAs was assessed using repeated-measures ANOVA followed by Tukey’s multiple comparisons test. *P < 0.05; **P < 0.01; ***P < 0.001, respectively.

Because the GLI1/GLI2 inhibitor GANT61 [20] ameliorated BM fibrosis in MF murine models [11], we sought to determine whether and how GLI1 contributes to the induction of BM fibrosis. To assess the effect of GLI1 inhibition on fibrocyte formation, we incubated MF patients’ or normal donors’ BM LDCs with increasing concentrations of GANT61 and assessed the capacity of those cells to form fibrocytes. We found that after 7 days of culture GANT61 inhibited the formation of MF and NC fibrocytes in a dose-dependent manner (Fig. 3A, B), suggesting that GLI1 is required for the differentiation of monocytes into fibrocytes.

Because GANT61 inhibited both MF and normal fibrocyte-forming cells which, unlike MF cells, do not express GLI1, we wondered whether the suppressive effect of GANT61 might not be GLI1-specific. Therefore, we used GLI1-siRNA to delineate the role of GLI1 in MF fibrocyte formation. Since under culture conditions that favor differentiation of monocytes into fibrocytes, cultured LDCs differentiate into fibrocytes or undergo apoptosis [21], we transfected MF BM LDCs with GLI1-siRNA, incubated them in culture conditions that favor fibrocyte formation and assessed their apoptosis rate by annexin V/propidium iodide (PI) staining using flow cytometry. Compared to transfection with scrambled control siRNA (Ctrl-siRNA), transfection with GLI1-siRNA induced a significantly higher apoptosis rate after 24 h of incubation (*P* < 0.001; Fig. 3C, D), suggesting that GLI1 knockdown induces apoptosis of MF fibrocyte-forming cells. Taken together, these data suggest that GLI1 is required for MF fibrocyte formation.

### GLI1 knockdown impedes MF fibrocyte viability

**Fig. 4:**
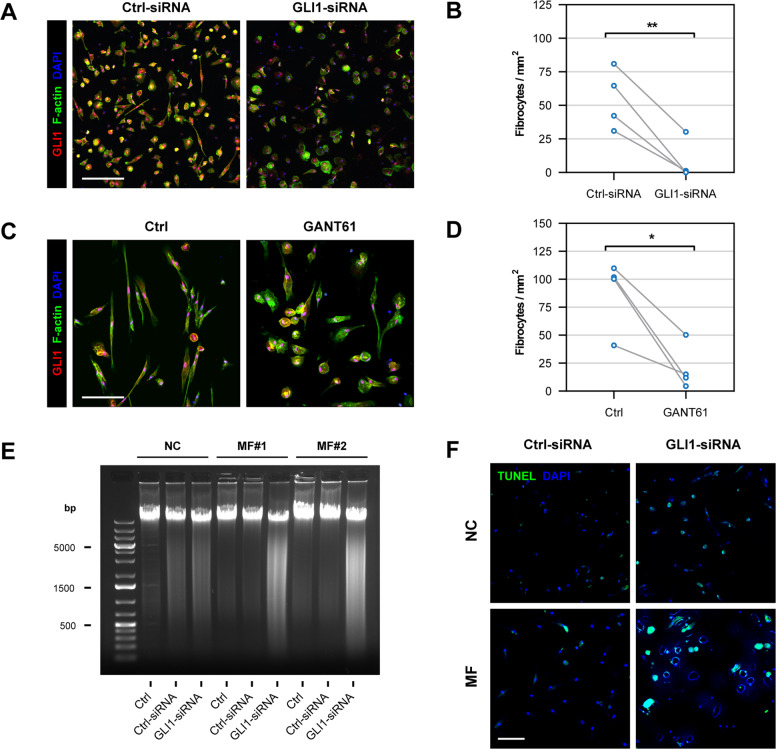
Inhibition of GLI1 induces apoptosis of MF fibrocytes. **A** Representative micrographs of MF BM LDC-derived fibrocytes transfected with GLI1-siRNA or Ctrl-siRNA were analyzed by fluorescence immunostaining. Fibrocytes were cultured from BM LDCs of MF patients and transfected with GLI1-siRNA for 48 h. Cells were stained for GLI1 (Alexa Fluor 594, pseudo-colored red) and F-actin (Alexa Fluor 488, pseudo-colored green) with DAPI (pseudo-colored blue) as a nuclear counterstain. **B** Quantitation of fibrocytes from MF patients (*n* = 4) following GLI1 silencing showed that GLI1-siRNA induced a significant reduction in fibrocyte counts. Fibrocyte counts were assessed by using an unsupervised cell segmentation algorithm, and the mean cell number per unit area was calculated for each well. The paired *t*-test assessed the statistical significance of differences between matched samples. **C** Representative micrographs of GANT61-treated MF BM LDC-derived fibrocytes analyzed by fluorescence immunostaining. Cultured viable and morphologically intact fibrocytes were incubated with 10 µM GANT61 for 72 h. Cells were stained for GLI1 (Alexa Fluor 594, pseudo-colored red) and F-actin (Alexa Fluor 488, pseudo-colored green) with DAPI (pseudo-colored blue) as a nuclear counterstain. **D** Quantitation of fibrocytes from MF patients (*n* = 4) following treatment with GANT61 showed that GANT61 significantly reduced fibrocyte numbers. An unsupervised cell segmentation algorithm determined fibrocyte numbers per unit area, and the means in each well were calculated. Matched values were compared using the paired *t*-test. **E** DNA laddering analysis of GLI1-silenced fibrocytes. Fibrocytes were cultured from BM LDCs of MF patients (*n* = 2) and BM LDCs of an NC and transfected with GLI1-siRNA or Ctrl-siRNA. Total genomic (g) DNA was isolated and 0.5 µg of gDNA was separated using agarose gel electrophoresis. DNA fragmentation observed in GLI1-silenced MF fibrocytes suggests that GLI1-siRNA, but not Ctrl-siRNA, significantly reduced fibrocyte viability. **F** The panel depicts representative micrographs of fibrocyte apoptosis rates assessed by TUNEL staining. High levels of biotinylated dNTPs (Alexa Fluor 488, pseudo-colored green) were detected in MF fibrocytes transfected with GLI1-siRNA. In contrast, biotinylated dNTPs were not detected in NC fibrocytes transfected with GLI1-siRNA or in MF fibrocytes transfected with Ctrl-siRNA. In addition, cell nuclei (stained with DAPI, pseudo-colored blue) show signs of karyorrhexis not observed in NC fibrocytes or fibrocytes transfected with Ctrl-siRNA. All micrographs were adjusted for brightness and contrast linearly and equally between groups. Scale bars represent 100 µm. *P < 0.05; **P < 0.01, respectively.

Because GLI1 knockdown induced apoptosis of MF fibrocyte-forming cells, we wondered whether inhibition of GLI1 would also alter the viability of mature fibrocytes. To test this hypothesis, we transfected mature MF BM-derived fibrocytes with GLI1-siRNA or incubated them with the GLI1/GLI2 inhibitor GANT61. We found that transfection with GLI1-siRNA reduced the number of viable fibrocytes by 6.9-fold (*P* < 0.01; Fig. 4A, B) 48 h after transfection. Similarly, incubation of mature fibrocytes with 10 μM GANT61 induced a 4.3-fold reduction in fibrocyte numbers (*P* < 0.05; Fig. 4C, D). Then, to determine whether the reduction in fibrocyte number is a result of apoptotic cell death, we used DNA laddering and terminal deoxynucleotidyl transferase dUTP nick-end labeling (TUNEL). Transfection with GLI1-siRNA but not Ctrl-siRNA, altered the viability of MF fibrocytes as assessed by bulk DNA laddering (Fig. 4E). Similarly, imaging analysis of individual fibrocytes labeled by TUNEL showed increased apoptosis rates of MF but not NC GLI1-siRNA-transfected fibrocytes (Fig. 4F), suggesting that GLI1 provides MF fibrocytes with survival advantage.

### GLI1 activates pro-fibrotic pathways in MF fibrocytes

**Fig. 5:**
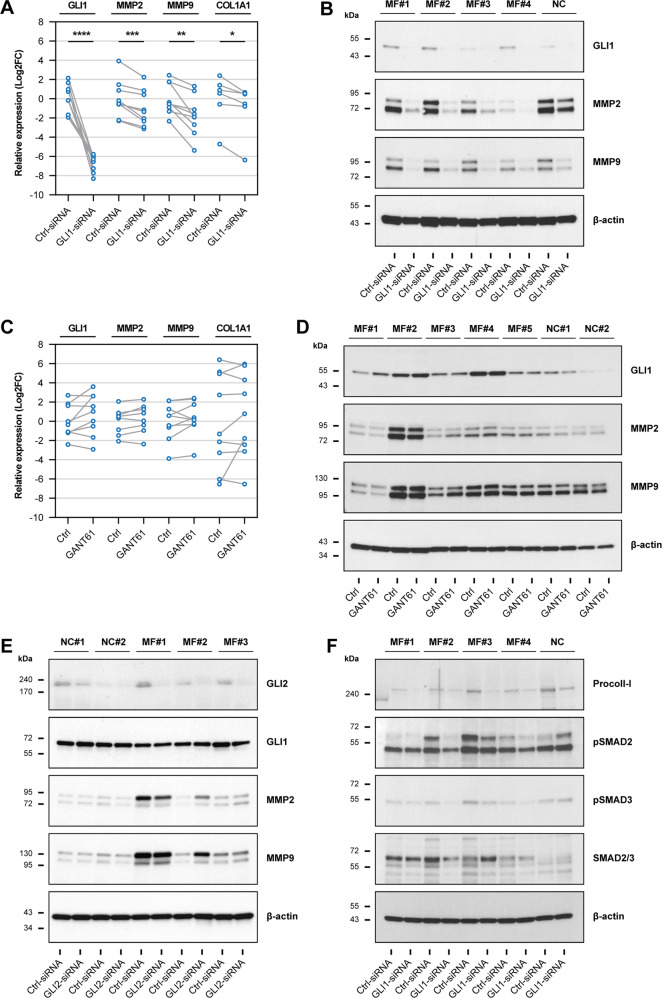
GLI1 activates pro-fibrotic signaling pathways in MF fibrocytes. **A**, **B** Quantitation of GLI1, MMP2, MMP9, and procollagen-I (*COL1A1*) levels in MF fibrocytes following GLI1 silencing. Cultured fibrocytes from MF patients were transfected with GLI1-siRNA or Ctrl-siRNA and expression analyses were performed 48 h following siRNA transfection. As shown, GLI1 silencing induced a significant reduction in GLI1, MMP2, and MMP9 mRNA (**A** *n* = 9) and protein levels (**B** *n* = 4) as assessed by qRT-PCR and western immunoblotting, respectively. **C**, **D** Quantitation of GLI1, MMP2, MMP9, and procollagen-I (*COL1A1*) levels in fibrocytes treated with GANT61. Cultured fibrocytes from MF patients were incubated with 10 µM GANT61 for 72 h or left untreated (Ctrl). Neither expression analyses of mRNA by qRT-PCR (**A** *n* = 9) nor protein by western immunoblotting (**B** *n* = 5) showed significant change in the levels of the analyzed genes following treatment with GANT61. **E** Protein levels of GLI2, GLI1, MMP2, and MMP9 in MF fibrocytes after GLI2 silencing. Cultured BM fibrocytes from MF patients (*n* = 3) and NCs (*n* = 2) were transfected with GLI2-siRNA or Ctrl-siRNA and expression analyses were performed 48 h following siRNA transfection. As shown by western immunoblotting, GLI2 silencing had no significant effect on GLI1 or its target genes. **F** Protein levels of procollagen-I, phosphorylated (p) and total SMAD2 and SMAD3 in GLI1-silenced fibrocytes were analyzed by western immunoblotting. Fibrocytes from MF patients (*n* = 4) and an NC were transfected with GLI1-siRNA or Ctrl-siRNA and their protein was collected after 48 h. As shown, GLI1 silencing reduced procollagen-I and phosphorylated SMAD2/3 levels. The PPIA gene was used as an internal control in the qRT-PCR analyses. Matched values were compared by using the paired *t*-test. All target proteins were detected by sequential probing of the same blot and β-actin was used as loading control. *P < 0.05; **P < 0.01; ***P < 0.001; ****P < 0.0001, respectively.

MF fibrocytes express high levels of GLI1 that, as shown above, protect fibrocytes from undergoing spontaneous apoptosis. However, whether or how GLI1 affects MF fibrocytes’ pro-fibrotic function remains obscure. Because MMPs activate TGF-β and promote collagen formation [22–25], we explored what effect GLI1 knockdown would have on GLI1-induced MMPs. We transfected MF fibrocytes with GLI1-siRNA and, by using qRT-PCR, found that GLI1-siRNA induced a significant reduction in the expression of MMP2, MMP9 and of procollagen-I (*COL1A1*) (Fig. 5A). Then, by using western immunoblotting we assessed MMP2 and MMP9 protein levels and found that transfection of MF fibrocytes with GLI1-siRNA reduced both MMP2 and MMP9 protein levels (Fig. 5B). Notably, the decrease in protein levels was more pronounced than in the corresponding mRNA levels suggesting that GLI1 in fibrocytes, in addition to inducing gene expression, promotes protein synthesis of MMPs via an indirect mechanism.

Remarkably, unlike GLI1-siRNA, GANT61 did not affect MMPs’ mRNA or protein levels (Fig. 5C, D), indicating that this compound lacks GLI1-mediated inhibitory effects in MF fibrocytes. To delineate this finding, we silenced the expression of GLI2 in BM fibrocytes by transfecting these cells with GLI2-siRNA. We found that, similar to GANT61, GLI2-siRNA had a negligible effect on protein levels of GLI1 and downstream MMPs (Fig. 5E), suggesting that GANT61 exerts its effect mainly through inhibition of GLI2.

Because MMP2 and MMP9 induce collagen formation by activating TGF-β [22–25], we used western immunoblotting to assess the effects of GLI1 silencing on the TGF-β downstream effectors SMAD2, SMAD3 and their phosphorylated (p) forms, and on procollagen-I protein levels. We found that GLI1-siRNA reduced pSMAD2, pSMAD3, and procollagen-I levels (Fig. 5F), suggesting that GLI1 activates the downstream signaling of TGF-β and enhances the production of collagen. Taken together, these data suggest that GLI1 activates pro-fibrotic pathways in MF fibrocytes.

### Constitutively activated STAT3 induces the expression of GLI1 in MF fibrocytes

**Fig. 6:**
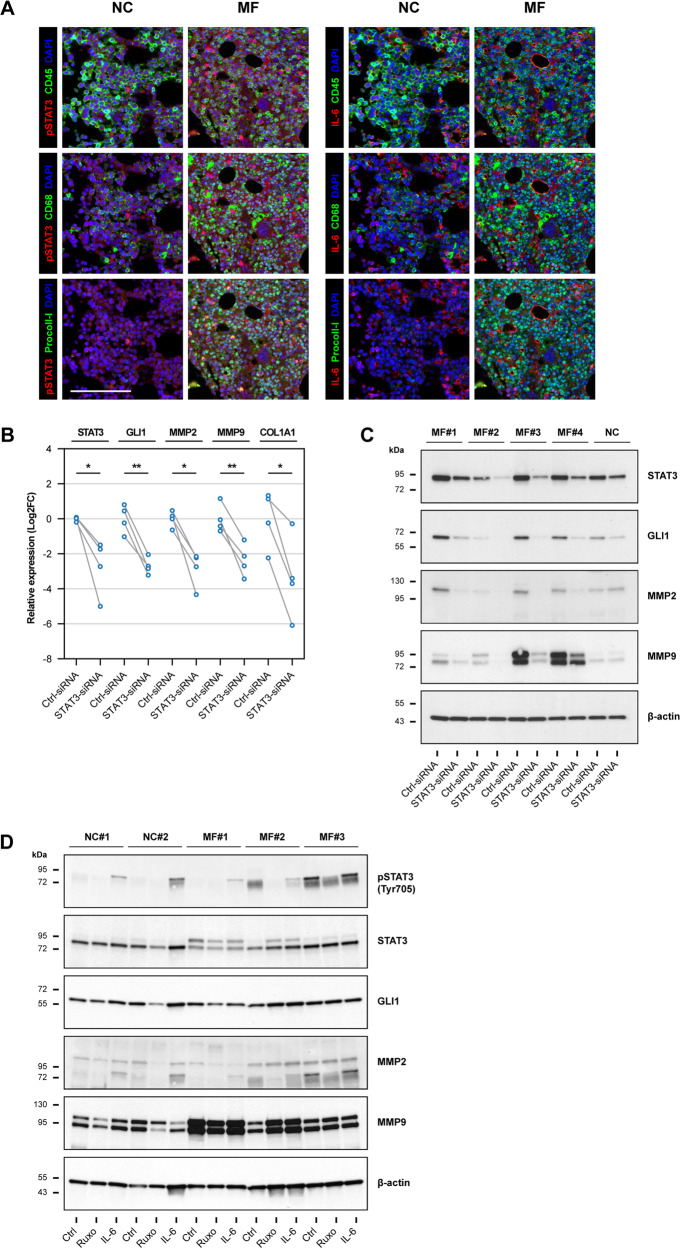
STAT3 is activated in MF fibrocytes and induces expression of GLI1. **A** Representative micrographs of phosphotyrosine (p) STAT3 and IL-6 expression in BM fibrocytes. BM tissue sections from MF patients (*n* = 6) and NCs (*n* = 4) were analyzed by mfIHC using pSTAT3 Tyr705 (Opal 480) and IL-6 antibodies (Opal 520) (pseudo-colored red) in combination with fibrocyte-specific antibodies CD45 (Opal 690), CD68 (Opal 620), and procollagen-I (Opal 570) (pseudo-colored green). Shown are composites of each fibrocyte marker with pSTAT3 (left panel) and IL-6 (right panel). As shown, pSTAT3 was detected in CD45^+^/CD68^+^/procollagen-I^+^ cells (fibrocytes) which are abundant in the BM of MF patients, but not NCs. IL-6 exhibited mostly extracellular localization and did not significantly differ between the two groups. DAPI (pseudo-colored blue) was used as a nuclear counterstain. All micrographs were adjusted for brightness and contrast linearly and equally between groups. The scale bar represents 100 µm. **B** Quantitation of STAT3, GLI1, MMP2, MMP9, and procollagen-I (*COL1A1*) mRNA levels in MF fibrocytes after STAT3 silencing. Cultured fibrocytes from MF patients (*n* = 4) were transfected with STAT3-siRNA. qRT-PCR analysis was conducted 48 h after transfection. All four analyzed genes were significantly downregulated following transfection with STAT3-siRNA as compared with transfection with Ctrl-siRNA. The panel depicts relative expression of mRNA normalized to mean mRNA levels in NC fibrocytes. Matched values were compared using the paired *t*-test. The PPIA gene was used as an internal control. **C** Protein levels of STAT3, GLI1, MMP2, and MMP9 in fibrocytes of MF patients (*n* = 4) and an NC after STAT3 silencing as analyzed by western immunoblotting. A significant decrease in all 4 analyzed proteins was detected in MF fibrocytes but was not detected in GLI1-target genes from NC fibrocytes. **D** Protein levels of pSTAT3 (Tyr705), STAT3, GLI1, MMP2, and MMP9 in MF fibrocytes after treatment with JAK1/2 inhibitor ruxolitinib (Ruxo) and IL-6. BM fibrocytes cultured from MF patients (*n* = 3) or NCs (*n* = 2) were incubated with 0.3 μM ruxolitinib, 100 ng/mL IL-6, or left untreated (Ctrl) for 24 h. Protein levels were then analyzed by western immunoblotting. Treatment with ruxolitinib reduced levels of pSTAT3, GLI1, and MMPs in most MF and NC fibrocyte cultures. In contrast, the addition of IL-6 enhanced the phosphorylation of STAT3 and increased the expression of GLI1 and its target genes. All target proteins were detected by sequential probing of the same blot. β-actin was used as the loading control. *P < 0.05; **P < 0.01, respectively.

Because we recently found that STAT3 induces the expression of GLI1 in chronic lymphocytic leukemia (CLL) and multiple myeloma cells [15], and because the JAK-STAT pathway is constitutively activated in MF [13, 14], we wondered whether STAT3upregulates GLI1 in MF fibrocytes. Using mfIHC we detected high levels of phosphotyrosine (p) STAT3 in BM biopsies of MF patients that co-localized with CD45^+^/CD68^+^/procollagen-I^+^ cells corresponding to fibrocytes (Fig. 6A, left panel). In contrast, we found no difference in the expression of a known STAT3 inducer interleukin (IL)-6 (Fig. 6A, right panel). Because we previously detected high levels of GLI1 in MF patients’ BM fibrocytes (Fig. 1), we reasoned that STAT3 induces the expression of GLI1 in these cells.

To test this assumption, we transfected the MF fibrocytes with STAT3-specific siRNA. Using qRT-PCR and western immunoblotting we found that STAT3 silencing significantly downregulated mRNA levels (Fig. 6B) and protein levels (Fig. 6C) of GLI1, MMP2, MMP9, and procollagen-I (*COL1A1*). Because JAK1/2 inhibitor ruxolitinib is an upstream inhibitor of STAT3 and IL-6 exerts its effects by inducing STAT3 [26, 27], we treated the MF fibrocytes with ruxolitinib and IL-6. Using western immunoblotting we found that upstream inhibition of STAT3 by ruxolitinib reduced, whereas STAT3 activation by IL-6 increased the levels of GLI1 and MMPs in fibrocytes from healthy donors and patients with MF (Fig. 6D), suggesting that GLI1 expression in MF fibrocytes is induced by constitutively activated STAT3.

### STAT3 transcriptionally activates the GLI1 gene in MF fibrocytes

**Fig. 7:**
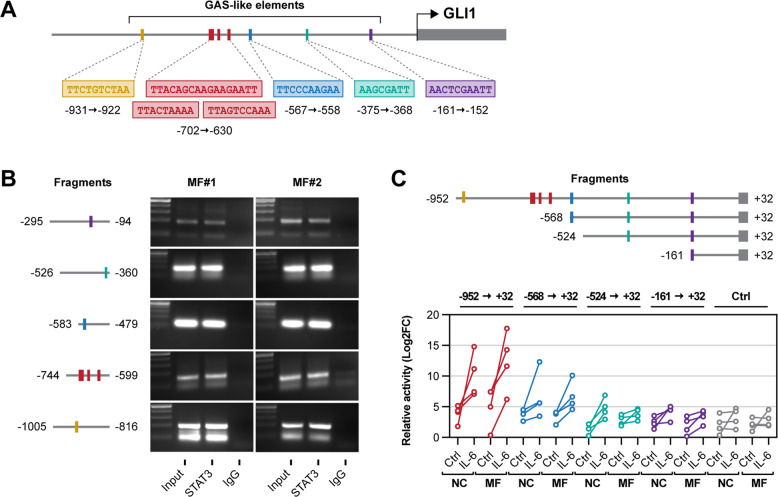
STAT3 binds to and activates the GLI1 gene promoter in MF fibrocytes. **A** Putative STAT3-binding sites in the GLI1 gene promoter region. GAS-like elements located within 1000 bp upstream of GLI1 gene start codon (*n* = 7) were identified by sequence analysis. **B** Chromatin immunoprecipitation analysis of STAT3-bound GLI1 gene promoter fragments from MF BM LDCs. Chromatin fragments from two representative MF patients were co-immunoprecipitated by anti-STAT3 antibodies or negative control IgG and GLI1 gene promoter fragments were amplified by PCR using five different primer sets, each including 1–3 GAS-like elements. Fragments were visualized using agarose gel electrophoresis. Co-immunoprecipitation with STAT3 was detected in all five analyzed fragments. **C** Quantitation of luciferase activity in MF fibrocytes transfected with luciferase reporter constructs containing GLI1 gene promoter fragments. Relative luciferase activity was measured in the presence or absence of IL-6, known to induce STAT3 phosphorylation. GLI1 gene promoter fragments harboring less GAS-like elements exhibited reduced luciferase activity. The relative luciferase activity was increased with the addition of IL-6, but not in fibrocytes transfected with negative control (Ctrl) constructs. Linear diagrams depict DNA base pair (bp) fragments upstream of the GLI1 start codon.

Because in CLL cells STAT3 directly binds to and activates the GLI1 gene promoter [15], we sought to determine whether STAT3 induces GLI1 expression in MF fibrocytes through a similar mechanism. We previously identified 7 gamma-interferon activated sequence (GAS)-like elements, considered putative STAT3-binding sites, within 1000 bp upstream of the GLI1 gene start codon (Fig. 7A and Supplementary Table S6). Using chromatin immunoprecipitation on BM LDCs isolated from two MF patients we found that, like in CLL cells [15], STAT3 co-immunoprecipitated DNA of all analyzed fragments, indicating that STAT3 binds the GLI1 gene promoter in MF fibrocyte-forming cells (Fig. 7B).

To validate this finding in MF fibrocytes, we performed the luciferase assay using four GLI1 gene promoter fragments with decreasing numbers of GAS-like elements. We incubated the cells with or without IL-6 to further activate STAT3 (Fig. 7C). We found that STAT3, although it is constitutively activated in MF, induced relative luciferase activity and that the addition of IL-6 further increased the luciferase activity in MF fibrocytes. The luciferase activity slightly decreased after each GAS-like element was truncated and the lowest luciferase activity was detected in fibrocytes transfected with the negative control construct, suggesting that STAT3 binds to these GAS-like elements and activates the GLI1 gene promoter in MF fibrocytes. Taken together, these data suggest that JAK2-induced constitutively activated STAT3 induces the expression of GLI1 in MF fibrocytes and likely in other MF neoplastic hematopoietic cells.

## Discussion

A recent study suggested that GLI1 levels in BM cells of patients with MF correlate with the degree of BM fibrosis [11, 12]. Hereby, we demonstrate that unlike MSCs, MF-derived fibrosis-inducing fibrocytes express high levels of GLI1 and that GLI1 provides MF fibrocytes with a survival advantage. We also show that JAK2-activated STAT3 induces the expression of GLI1 and that GLI1 activates pro-fibrotic signaling pathways in fibrocytes of patients with MF.

The Hh pathway plays a critical role in embryonic development [10]. Although dormant in adult cells, the Hh pathway is often reactivated in various neoplasms and acts as a potent oncogenic regulator [28, 29]. Several investigators reported reactivation of the Hh signaling pathway and its downstream effectors, such as GLI1, in hematologic neoplasms including MPN [15, 30–34]. Whereas we found that GLI1 is overexpressed in MF patients’ BM LDCs, other investigators detected GLI1 in MPN patients’ granulocytes [33], but not in their circulating LDCs [35]. Nevertheless, in agreement with our findings, high levels of the Hh pathway genes were detected in the BM and spleen of an MF mouse model [36].

Because GLI1 was detected in neoplastic cells expressing activated STAT3 [15, 37–39], we performed in situ imaging analysis of BM biopsies and confirmed that MF fibrocytes harbor phosphotyrosine STAT3. In patients with MF JAK2 is constitutively activated and induces the phosphorylation of STAT3 [13, 14]. Therefore, we reasoned that STAT3 would induce the expression of GLI1 in these cells. As expected, we found that STAT3 silencing and inhibition downregulated GLI1, whereas STAT3 activation by IL-6 upregulated GLI1. However, STAT3-induced GLI1 expression was not consistent across the examined patients, suggesting that MF fibrocytes have varying degrees of constitutive STAT3 activity and sensitivity to STAT3 inhibition. Recently, sequence analysis of the GLI1 gene revealed that the GLI1 gene promoter harbors STAT3-binding sites [15]. Therefore, we sought to assess the STAT3-mediated activation of the GLI1 gene in MF cells. We found that, like in CLL and breast cancer [15, 38], in MF fibrocytes and LDCs phosphorylated STAT3 binds to and activates the GLI1 gene promoter.

Fibrocytes are distinct hematopoietic cells known to share several features with MSCs. Like MSCs, fibrocytes produce collagen-I, collagen-III, and fibronectin [17, 18, 40]. We and others demonstrated that in patients with MF neoplastic monocyte-derived fibrocytes are the significant contributors to the induction of BM fibrosis [6–8, 41]. GLI1 plays a central role in the regulation of embryonic, but not adult, cell development and in neoplastic cell oncogenesis [9]. Mouse model studies revealed that GLI1 has no effect on normal hematopoietic cell self-renewal or differentiation [42, 43]. In agreement with these reports we found that, unlike healthy donors’ fibrocytes, MF patients’ fibrocytes overexpress GLI1 that promotes the differentiation of fibrocyte progenitors into mature fibrocytes. Furthermore, we found that GLI1 exerts an anti-apoptotic effect on MF fibrocytes and, as previously described [22–24], triggers pro-fibrotic signaling pathways by activating the downstream TGF-β effectors SMAD2 and SMAD3, and by inducing the expression of procollagen-I.

Reactivation of the Hh pathway and upregulation of GLI1 expression have been observed in conditions with fibrocyte-induced tissue fibrosis [17], such as idiopathic pulmonary fibrosis [44], biliary fibrosis [45], renal fibrosis [46], and cardiac fibrosis [47]. Like in MF [11], GLI1 contributed to the induction of various forms of fibrosis by MSC-like cells in murine models [48]. It is therefore possible that, similar to BM fibrosis of patients with MF, GLI1 plays a role in fibrocyte-induced fibrosis of other tissues perhaps by exogenous activation of STAT3.

In MF clinical trials, Hh inhibitors exerted limited therapeutic benefits [49]. Although the Smoothened inhibitor saridegib induced a modest reduction in spleen size and the degree of BM fibrosis, it did not reduce GLI1 mRNA or protein levels [50]. By contrast, the Hh inhibitor sonidegib reduced GLI1 levels when combined with the JAK1/2 inhibitor ruxolitinib [33, 51], suggesting that GLI1 expression in MF is induced by both Hh pathway and the JAK-STAT pathway.

Taken together, our data suggest that GLI1 plays a key role in the induction of BM fibrosis in MF by providing neoplastic fibrocytes with survival advantage and enhancing their pro-fibrotic function. Whether targeting GLI1 might alleviate BM fibrosis in patients with MF remains to be determined.

## Materials and methods

### Specimen collection and preparation

Trephine BM biopsies and BM aspirates were obtained from patients with PMF, post-ET, and post-PV MF who were treated at The University of Texas MD Anderson Cancer Center between May 2007 and Februrary 2022 and from hematologically normal individuals. The diagnosis of MF was established according to the 2008 World Health Organization revised criteria [1]. Control BM biopsies were obtained from morphologically normal BM donors and control BM aspirates were obtained from commercially available healthy donors (AllCells, Alameda, CA, USA). The patients’ and control BM donors’ clinical characteristics are depicted in Supplementary Tables S1 and S2, respectively. The median age difference between the two groups was 24 years; however, the exact age-matched controls were unavailable for most patients due to increased age. Sample sizes were not statistically determined, rather they were chosen based on specimen availability, similar to those commonly employed in the field. No samples were excluded from the analysis.

To prepare BM tissue sections, BM biopsies were fixed using 10% neutral buffered formalin, decalcified using 10% formic acid (Sigma-Aldrich, St. Louis, MO, USA), paraffin-embedded, and cut at a thickness of 4–6 µm. To isolate BM LDCs, BM aspirates were fractionated by density gradient centrifugation in Ficoll-Hypaque 1.077 g/mL medium (Sigma-Aldrich) and cells were recovered from the “buffy coat” layer.

### Fibrocyte and MSC culture assays

MF patients’ or normal donors’ BM LDCs were cultured in conditions favoring differentiation to either fibrocytes or MSCs, as previously described [6, 21, 52]. To differentiate LDCs to fibrocytes BM LDCs were cultured in StemSpan serum-free medium (Stemcell Technologies, Vancouver, BC, Canada) supplemented with modified eagle medium (MEM), non-essential amino acid, insulin-transferrin-selenium, HEPES, glutamine, sodium pyruvate, penicillin, and streptomycin solutions (Sigma-Aldrich). To grow MSCs, BM LDCs were cultured in α-MEM supplemented with 20% FBS (Invitrogen, Waltham, MA, USA). All LDC-derived cultures were grown in Nunc Lab-Tek II CC2 chamber slides (Thermo Scientific, Waltham, MA, USA) at 37 °C in humidified atmosphere supplemented with 5% CO_2_.

### Xenograft mouse model

BM LDCs (2 × 10^6^) aspirated from two different patients with MF were injected into the tail vein of 6–8- week old female NSG mice (Jackson Laboratories, Bar Arbor, ME, USA) and engraftment was assessed as previously described [6, 19]. Thirty-two weeks after transplantation, the animals were euthanized using CO_2_ and their sternum and spleen were harvested and processed. Sternum BM sections were submitted to Gomori’s silver stain to assess the levels of reticulin fibrosis. All animal studies were conducted at The University of Texas MD Anderson Cancer Center under a research protocol approved by the Institutional Animal Care and Use Committee. Institutional policies were adhered to in order to minimize animal suffering. The sample size was determined by power analysis that ensured a power of 80% and a two-sided significance level of 0.05 to detect estimated effect size of 1.7. No randomization or blinding method was used for group allocation during in vivo experiments.

### mfIHC staining

To simultaneously detect GLI1 and cell phenotype markers a mfIHC was performed using tyramide signal amplification approach as previously reported [19, 53]. Briefly, human BM tissue sections dried at 60 °C were deparaffinized and rehydrated in xylene-ethanol gradient, fixed with 3% hydrogen peroxide/methanol solution, and put through five or six rounds of staining. Each round consisted of antigen retrieval at 95 °C using Citra Plus in an EZ-Retriever system (BioGenex, Fremont, CA, USA), block with 3% bovine serum albumin, incubation with primary antibody (Supplementary Table S3, human panel A), and detection using SuperPicture broad-spectrum horseradish peroxidase polymer antibodies (Invitrogen) and an Opal fluorophore (Supplementary Table S5). DAPI (4′,6-diamidino-2-phenylindole; Invitrogen) was used as a nuclear counterstain. Xenograft mouse and human panel B tissue sections were stained by adapting the above protocol on a NanoVIP automated staining system (BioGenex). Deparaffination and antigen retrieval were performed using X-DeWax and EZ-AR1 or EZ-AR2 solutions (BioGenex). Blocking and detection of the primary antibodies were achieved using 2.5% goat serum solution and ImmPRESS species-specific horseradish peroxidase polymer antibodies (Vector Biolabs, Malvern, PA, USA), respectively.

### GLI1 immunostaining

Cultured fibrocytes or MSCs were fixed in 4% paraformaldehyde, blocked in a 3% (wt/v) bovine serum albumin/PBS solution, incubated with anti-GLI1 primary antibody overnight at 4 °C, followed by incubation with donkey anti-rabbit secondary antibody (Invitrogen). The phalloidin dye (Invitrogen) was used to detect F-actin and DAPI was used as a nuclear counterstain.

### Multiplexed fluorescence RNA in situ hybridization

Cultured fibrocytes’ mRNA levels were assessed by RNA in situ hybridization using the RNAscope Multiplex Fluorescent V2 assay and HybEZ II hybridization system (Advanced Cell Diagnostics, Newark, CA, USA). Cultured fibrocytes fixed with 4% paraformaldehyde were dehydrated using ethanol gradient and, after rehydration and treatment with hydrogen peroxide and Protease III solutions, hybridized with mRNA-specific probes for 2 h at 40 °C. Then, cultured fibrocytes were exposed to multiple rounds of signal amplification that resulted in the binding of a different Opal fluorophore to each probe (Supplementary Tables S4, S5). Probes targeting housekeeping genes *POLR2A*, *PPIB*, *UBC*, and *HPRT1* were used as positive controls and the probe targeting bacterial *dapB* gene was used as a negative control.

### Gene expression analysis

To quantitate gene expression levels, we used qRT-PCR. Briefly, cellular RNA was extracted using a Direct-zol RNA Microprep kit (Zymo Research, Irvine, CA, USA). Extracted RNA concentration was determined using ND1000 spectrophotometer (NanoDrop Technologies, Wilmington, DE, USA) and 0.5 µg of the total RNA were amplified by real-time PCR using Superscript First-Strand III synthesis supermix (Invitrogen). The FAM-labeled TaqMan minor groove binder probes (Supplementary Table S4) and TaqMan Universal PCR master mix on QuantStudio 7 Flex platform (Applied Biosystems, Waltham, MA, USA) were used for quantitation. Relative expression levels were calculated by subtracting cycle threshold value of a housekeeping gene (*PPIA*) from the cycle threshold value of each target gene.

### Western immunoblotting

To perform western immunoblotting, cells were lysed using RIPA lysis buffer (Thermo Scientific) and protein concentrations were assessed using a modified Lowry method (DC Protein Assay; Bio-Rad, Hercules, CA, USA). Cell lysates were loaded onto a 4–15% Criterion TGX Precast gel (Bio-Rad) and protein were transferred onto an Immobilon-P PVDF membrane (EMD Millipore, Burlington, MA, USA). The membranes were blocked with 5% (wt/v) non-fat milk/PBS-Tween20 (Sigma-Aldrich) solution and incubated with primary antibodies overnight at 4 °C (Supplementary Table S3). As secondary antibodies, anti-mouse or anti-rabbit horseradish peroxidase polymer antibodies were used (GE Healthcare, Chicago, IL, USA). Beta-actin served as a loading control. Bands were visualized using Amersham enhanced chemiluminescence detection reagents (GE Healthcare). All full-length original western immunoblots used in the study are provided in Supplementary material 2.

### Cell treatment

To inhibit GLI1, BM LDCs or mature fibrocytes were incubated in the absence or presence of increasing concentrations of the GANT61 (G9048; Sigma-Aldrich). To suppress STAT3 phosphorylation fibrocytes were incubated with JAK1/2 inhibitor ruxolitinib (R-6688; LC Laboratories, Woburn, MA, USA). To stimulate STAT3 phosphorylation, cells were incubated with HEK293 cell-derived recombinant IL-6 (7270-IL; R&D Systems, Minneapolis, MN, USA).

### Transfection with siRNA

To assess the effects of GLI1 or STAT3 gene silencing, siRNA was used (Supplementary Table S4). The transfection medium was prepared by adding 20 mM of siRNA to Lipofectamine RNAiMAX transfection reagent (Invitrogen) diluted in Opti-MEM reduced serum medium (Gibco, Waltham, MA, USA). Then, BM LDCs or mature fibrocytes were transfected with GLI1-, GLI2-, or STAT3-siRNA by adding the transfection medium in a 1:1 ratio and incubating the cells for 48 h at 37 °C. Ctrl-siRNA was used as a negative control.

### Annexin V apoptosis assay

To assess BM LDC apoptosis rates, we used the FITC Annexin V Apoptosis Detection Kit II (BD Biosciences, San Jose, CA, USA). Briefly, 1 × 10^6^ of cells washed with PBS were re-suspended in binding buffer supplemented with annexin V and PI solutions and analyzed by FACScan flow cytometer using the CellQuest Pro software (BD Biosciences). The cellular apoptosis rate was calculated as the percentage of annexin V-positive cells within the total BM LDC gate.

### DNA fragmentation assays

To assess fibrocyte apoptosis rates we used DNA laddering and TUNEL. Fibrocyte DNA was isolated using cell lysis and protein precipitation solutions (Qiagen, Germantown, MD, USA) and separated by electrophoresis on ethidium bromide-infused 0.8% agarose gel with a GeneRuler 1 kb Plus DNA ladder (Thermo Scientific) as reference. A modified TUNEL assay was conducted by using the TACS 2 TdT Fluorescein kit (Trevigen, Gaithersburg, MD, USA). Following fixation in 4% paraformaldehyde, fibrocytes were permeabilized with Cytonin reagent and incubated in labeling buffer supplemented with terminal deoxynucleotidyl transferase, biotinylated dNTPs, and cobalt cations. DNA fragments were detected using fluorescein-conjugated streptavidin and DAPI was used as a nuclear counterstain.

### Chromatin immunoprecipitation assay

The SimpleChIP Enzymatic Chromatin IP kit (Cell Signaling Technology, Boston, MA, USA) was used as previously described [15]. To obtain chromatin fragments, BM LDCs were crosslinked in 1% formaldehyde and lysed chromatin was partially digested using micrococcal nuclease and sonicated. Next, chromatin fragments were incubated with anti-STAT3 antibodies or IgG (Supplementary Table S3) overnight at 4 °C with protein G agarose beads. Chromatin fragments were eluted from antibody-bound protein and digested using proteinase K, and DNA was purified using spin columns. GLI1 gene promoter fragments amplified by PCR using specific primers (Supplementary Table S7) were detected by electrophoresis on an ethidium bromide-infused 2% agarose gel.

### Luciferase assay

A modification of a previously described luciferase assay was used [15]. In brief, interleukin (IL)-6-stimulated and -unstimulated MF fibrocytes were transfected with luciferase reporter constructs that contained GLI1 gene promoter fragments. Reporter constructs were generated using pGL4.17[*luc2*/Neo] vector (Promega, Madison, WI, USA) and primers listed in Supplementary Table S8. The pRL-SV40 vector producing Renilla luciferase was used as an internal control. The constructs were transfected into fibrocytes using a lipofectamine transfection medium and incubated for 24 h at 37 °C. Following transfection, the fibrocytes were incubated in 2% FBS/RPMI-1640 starvation medium (Gibco) for 1 h at 37 °C and IL-6 (20 ng/mL) was added. Detection of luciferase activity was performed using the Dual-Glo system (Promega, Madison, WI, USA) and luminescence levels were measured using Monolight 3010 luminometer (BD Biosciences). The relative luciferase activity was calculated by normalizing the firefly luminescence to the Renilla luminescence.

### Imaging analysis

Whole fluorescently labeled BM tissue sections were imaged using the Vectra Polaris imaging system (Akoya Biosciences, Menlo Park, CA, USA) with a spectral library built using single-stained and unstained reference slides. Images were spectrally unmixed and single-cell GLI1 expression was quantitated using a tissue- and cell-segmentation algorithm developed in the inForm software v2.2.1 (Akoya Biosciences). To quantitate GLI1^+^ fibrocytes we used a segmentation and phenotype algorithm developed in the VIS software v2020.04 (Visiopharm, Hørsholm, Denmark). The cultured cells were imaged using Andor Revolution XDi WD spinning disk confocal system with Zyla 4.2 sCMOS camera (Andor Technology, Belfast, UK) and UPlanSApo 30XS silicone oil-immersion objective lens (Olympus, Tokyo, Japan), and analyzed using Imaris v9.1.0 software (Bitplane, Zurich, Switzerland). To quantitate cultured fibrocytes we imaged entire wells in duplicates using the Vectra Polaris imaging system and performed cell segmentation using an algorithm developed in the VIS software.

### Statistical analysis

The statistical significance of the difference between groups was assessed using the one-way ANOVA, the repeated-measures ANOVA, the Mann–Whitney test, or the paired *t*-test, as appropriate. Post hoc multiple comparisons were performed using Tukey’s or Dunn’s method. Calculations were performed and data were visualized using GraphPad Prism software v9.0.0 (GraphPad Software, San Diego, CA, USA). Data within each group met test assumptions of normality and equal variances. All tests were performed two-sided and a *P* value of <0.05 was considered statistically significant.

## Supplementary information

Supplementary material 2

Supplementary material 1

## Data Availability

The authors will provide the data generated and analyzed in this study upon reasonable request to the corresponding author.
