# Supplementary material for: GLI1 activates pro-fibrotic pathways in myelofibrosis fibrocytes
Source: Cell Death Dis. 2022 May 20;13(5):481. doi: 10.1038/s41419-022-04932-4 (PMC9122946; doi:10.1038/s41419-022-04932-4)

Supplementary material 2

Full-length original western immunoblots

Fig. 1G

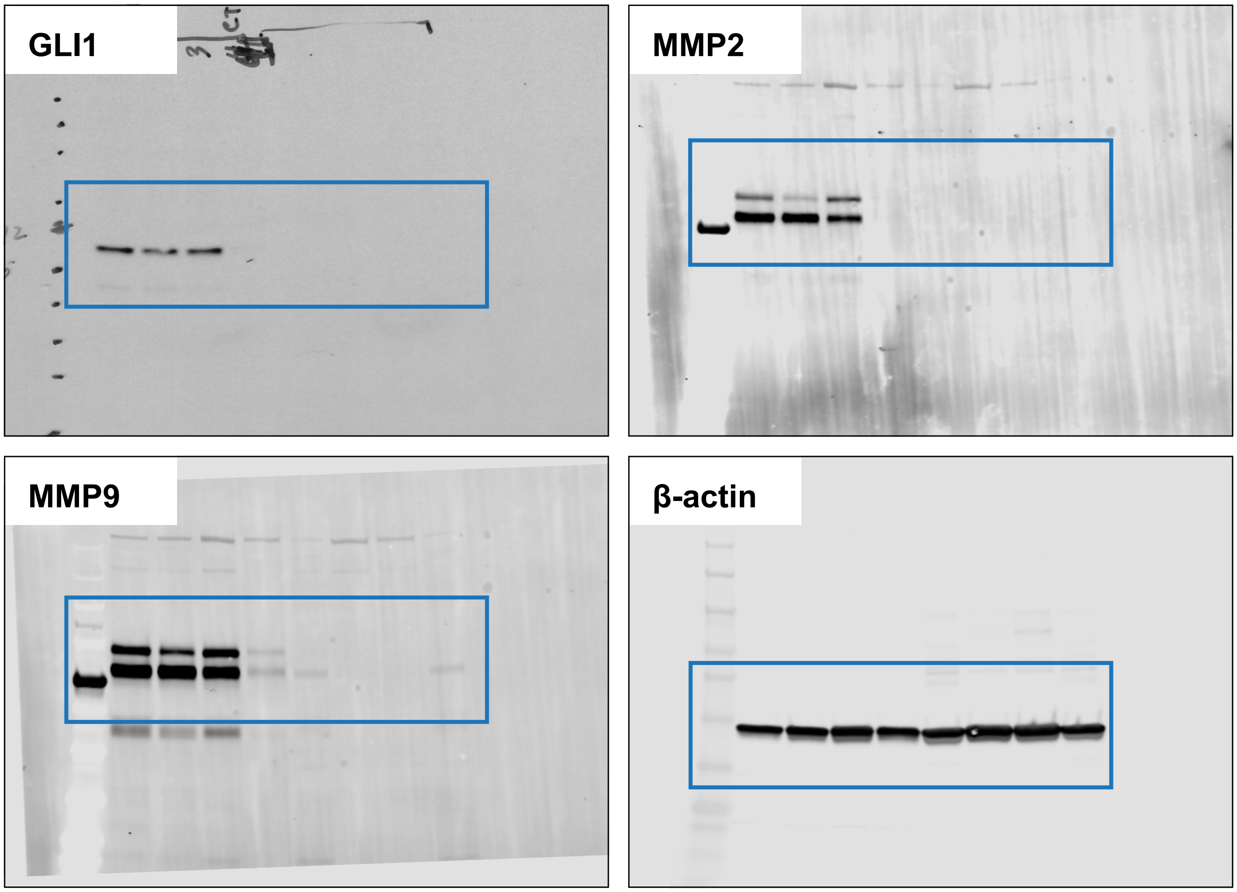

Fig. 5B

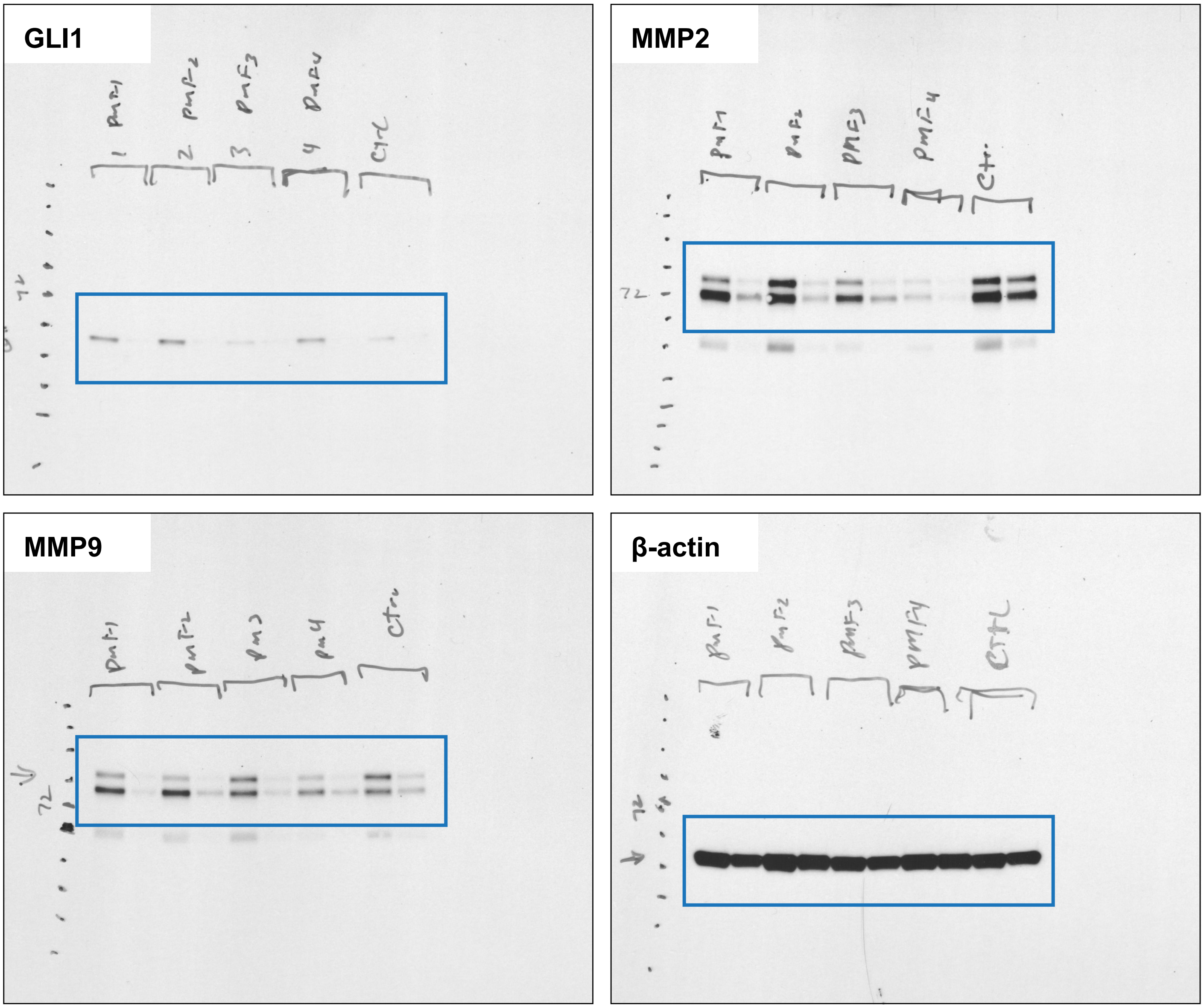

Fig. 5D

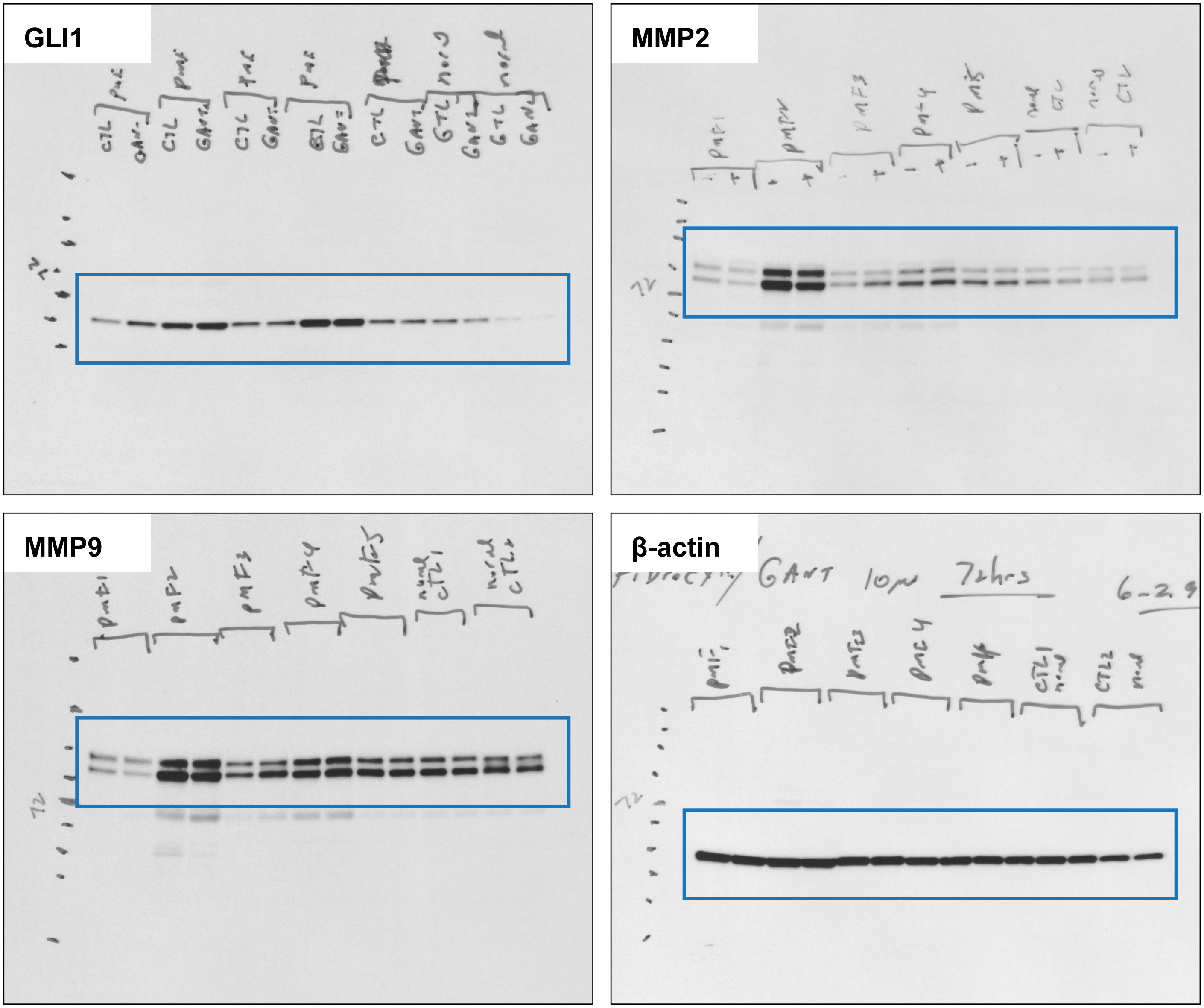

Fig. 5E

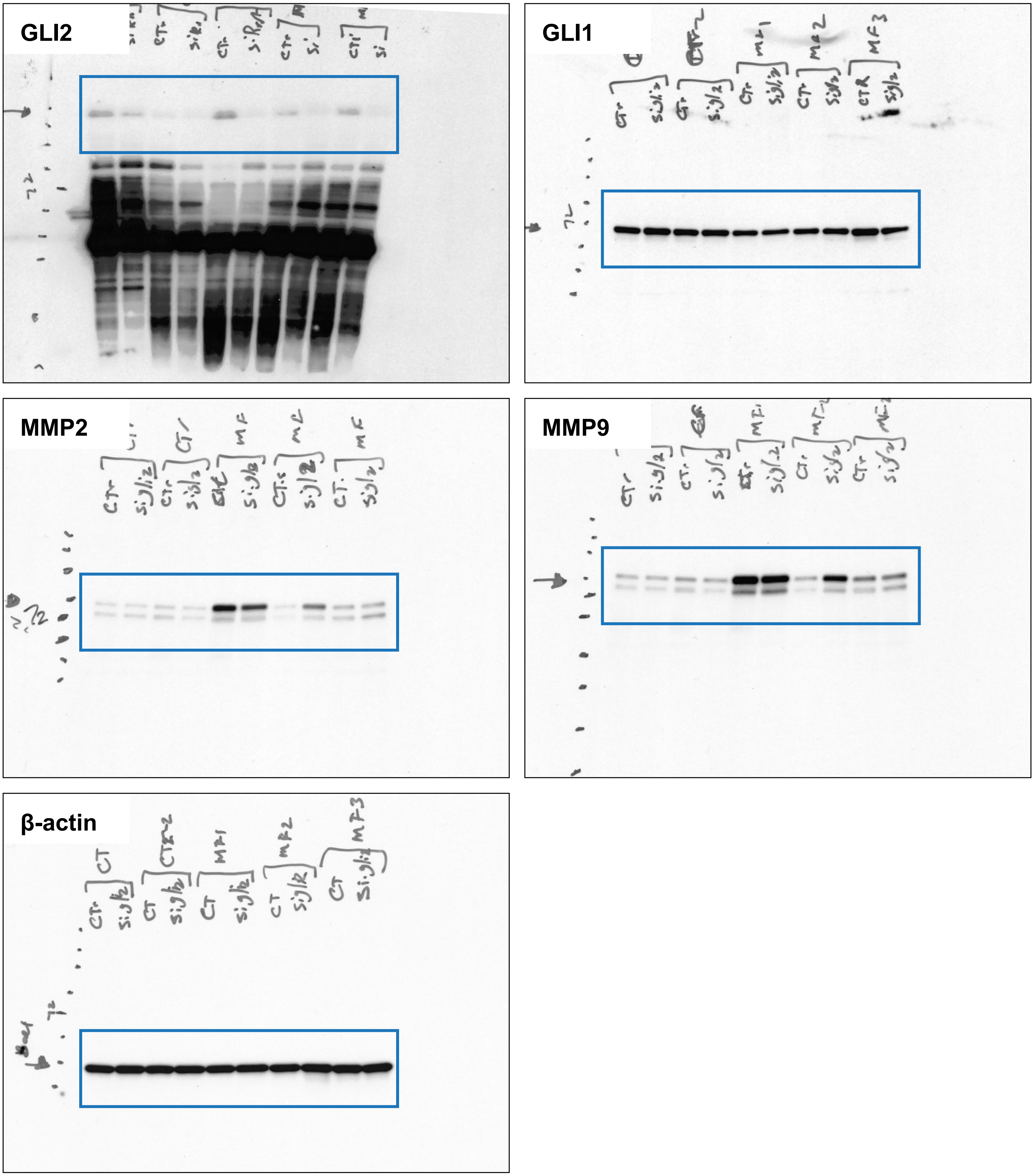

Fig. 5F

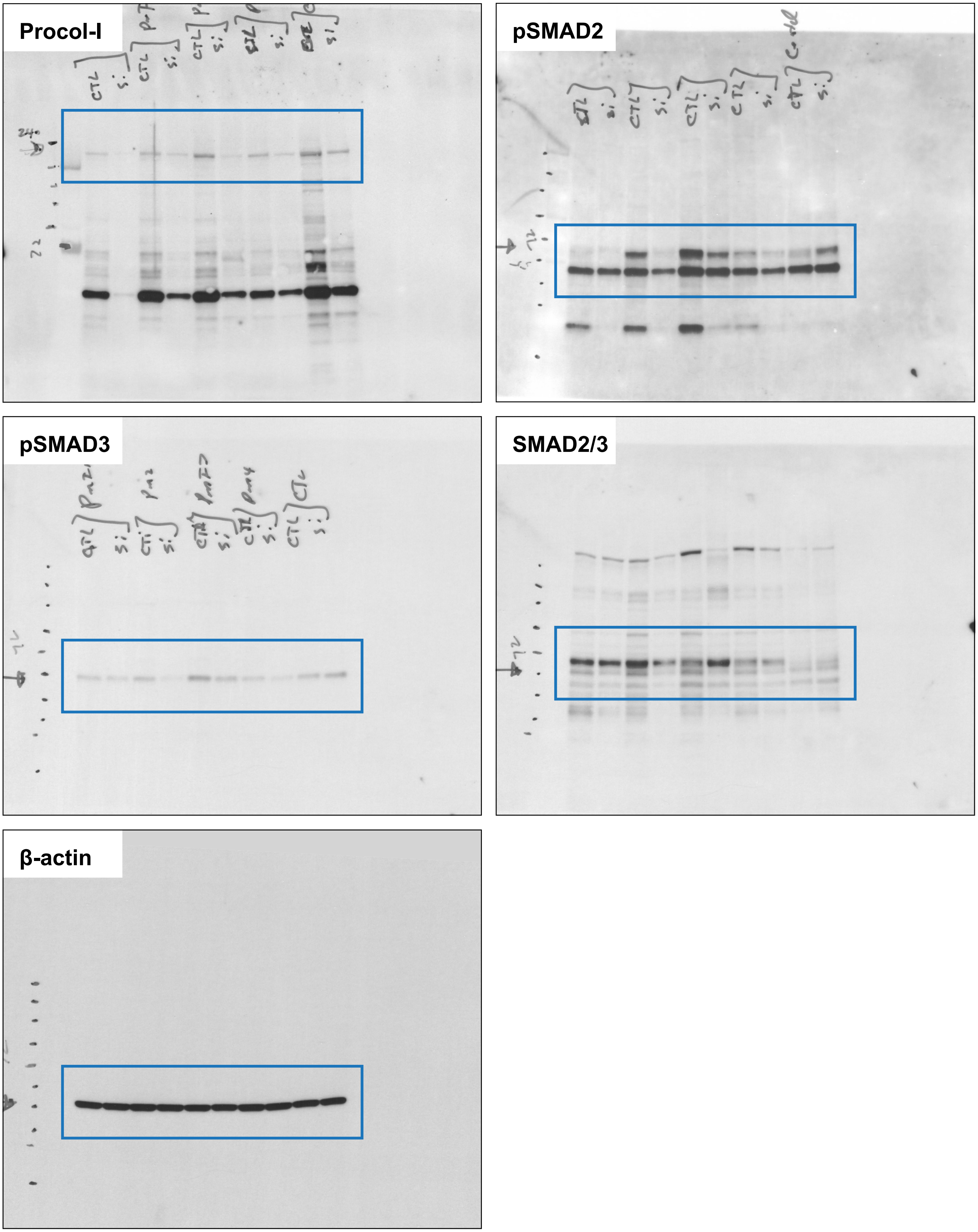

Fig. 6C

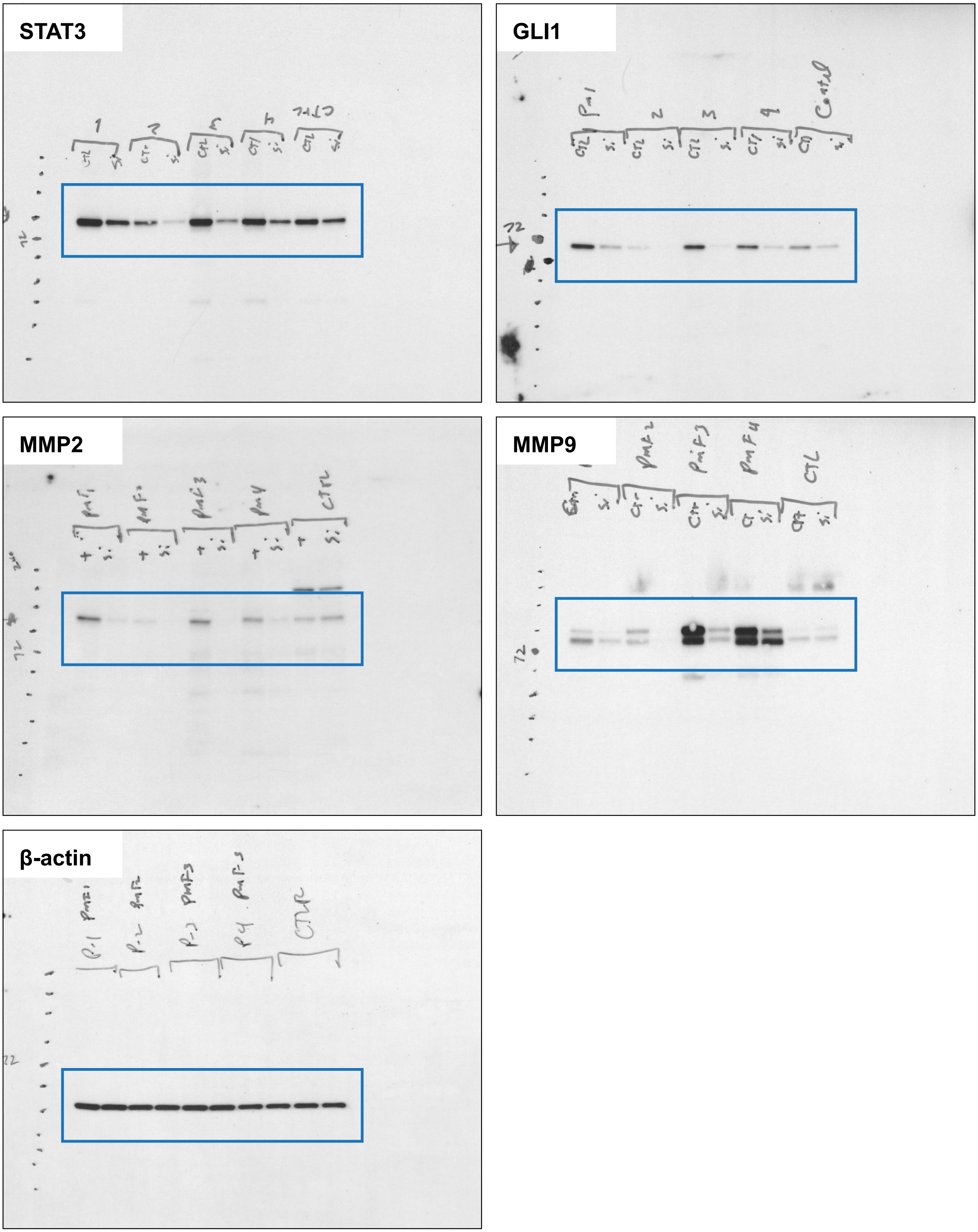

Fig. 6D

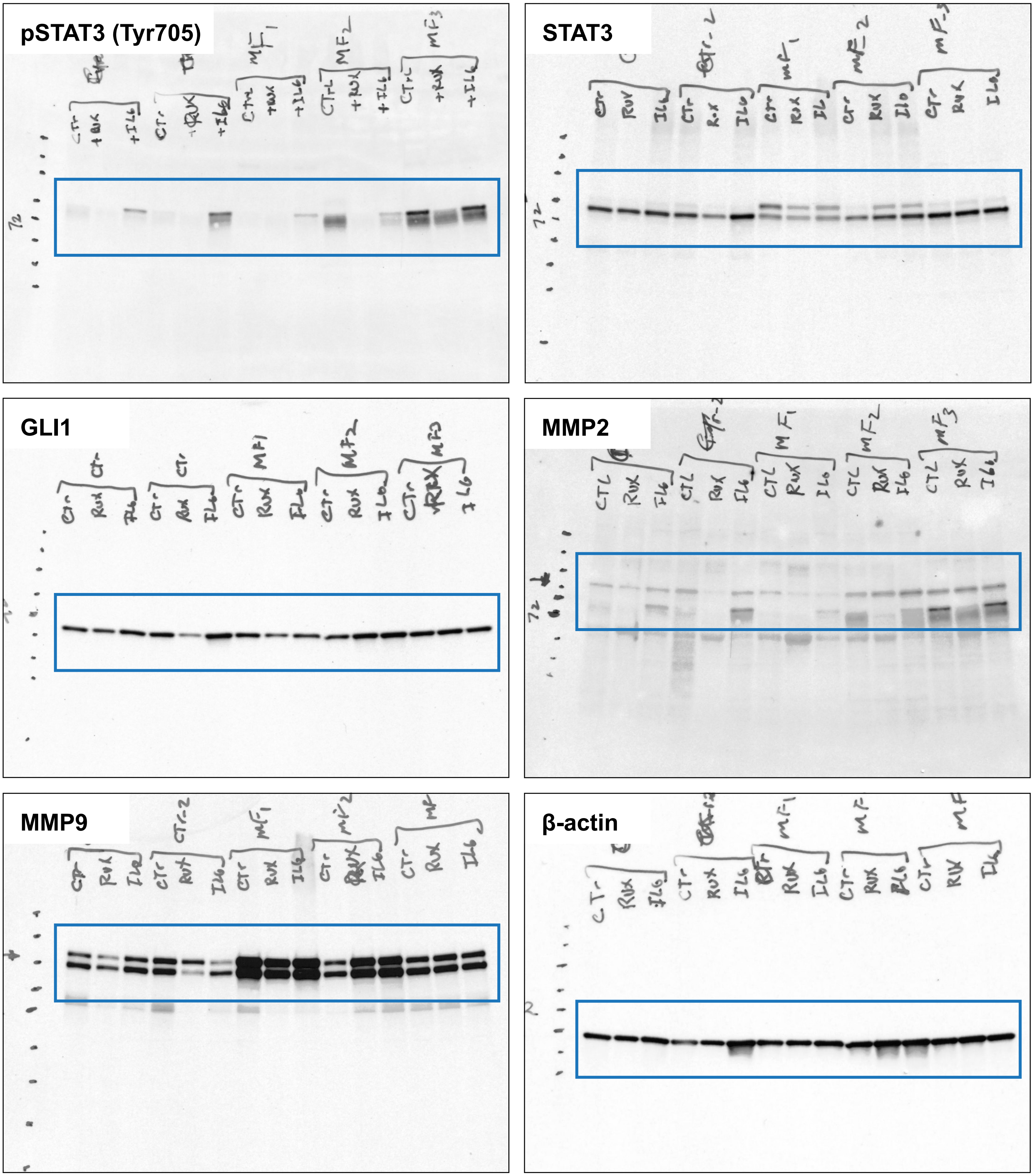

Supplement: Supplementary file 1 — Supplementary material 2 [file 41419_2022_4932_MOESM1_ESM.pdf]
