# Supplementary material for: GLI1 activates pro-fibrotic pathways in myelofibrosis fibrocytes
Source: Cell Death Dis. 2022 May 20;13(5):481. doi: 10.1038/s41419-022-04932-4 (PMC9122946; doi:10.1038/s41419-022-04932-4)
Supplement: Supplementary file 2 — Supplementary material 1 [file 41419_2022_4932_MOESM2_ESM.pdf]

Supplementary material 1

Supplementary Table S1. Patients' characteristics

Parameter	Median [range] *or* n (%)	Parameter	Median [range] *or* n (%)
Total patients	85 (100)		
**Demographics**		**Active treatment**	
Age (years)	65 (22 – 86)	None	33 (38.8)
Sex: Male	38 (44.7)	Ruxolitinib	25 (29.4)
**Diagnosis***		Hydroxyurea	14 (16.5)
Primary MF	48 (56.5)	Investigational drug	9 (10.6)
Post-ET MF	28 (32.9)	Anagrelide	5 (5.9)
Post-PV MF	9 (10.6)	IFN-α	2 (2.4)
**Bone marrow fibrosis grade†**		Azacitidine	1 (1.2)
MF-1	29 (34.1)	Thalidomide	1 (1.2)
MF-2	34 (40)	**Risk category‡**	
MF-3	22 (25.9)	Very low risk	3 (6)
**Driver mutation**		Low risk	17 (34)
JAK2 (V617F)	51 (67.1)	Intermediate risk	10 (20)
CALR (exon 9)	20 (26.3)	High risk	16 (32)
MPL (W515L)	4 (5.3)	Very high risk	4 (8)
Triple negative	1 (1.3)	**Peripheral blood counts**	
**Clinical and cytogenetic features**		Leukocytes > 25 $\times 10^9$/L	9 (10.6)
Time since diagnosis (months)	23 (0 – 224)	Hemoglobin < 10 g/dL	25 (29.4)
Splenomegaly§	20 (24.1)	Platelets < 100 $\times 10^9$/L	14 (16.5)
Constitutional symptoms‖	18 (21.2)	Blasts ≥ 2%	15 (17.6)
Unfavorable/VHR karyotype¶	14 (17.3)	Monocytes ≥ 8%	35 (41.2)

Abbreviations: MF, myelofibrosis; ET, essential thrombocythemia; PV, polycythemia vera; VHR, very high risk.
*Diagnosis was established according to the 2008 World Health Organization revised classification [1].
†Bone marrow (BM) fibrosis grade was determined using the European consensus criteria [2].
‡Risk categories were estimated using mutation and karyotype-enhanced prognostic scoring system (MIPSS) 70+, version 2.0 [3].
§Splenomegaly was defined as spleen palpable at ≥ 5 cm below the left costal margin.
‖Constitutional symptoms were defined as unexpected weight loss > 10%, unexplained fever, and/or excessive sweats persisting for more than 1 month.
¶Karyotypes were categorized based on revised cytogenetic risk stratification [4].

Supplementary Table S2. Bone marrow donors' characteristics

**Parameter**	**Median [range] *or* n (%)**
Total bone marrow donors	25 (100)
**Demographics**	
Age (years)	41 (24 – 70)
Sex: Male	12 (48)

Supplementary Table S3. Antibodies used in immunodetection assays

Target	Clone	Manufacturer	Catalog no.	RRID
**mfIHC, human panel A**				
GLI1	*Polyclonal*	Novus	NBP1-78259	AB_11030198
CD45	MEM-28	Abcam	ab8216	AB_306361
CD68	KP1	Abcam	ab955	AB_307338
Procollagen-I	M-58	EMD Millipore	MAB1912	AB_94405
CD90	EPR3132	Abcam	ab92574	AB_10563647
CD105	3A9	Cell Signaling Technology	14606	AB_2798534
**mfIHC, human panel B**				
pSTAT3 (Tyr705)	D3A7	Cell Signaling Technology	9145	AB_2491009
IL-6	1.2-2B11-2G10	Abcam	ab9324	AB_734850
CD45	MEM-28	Abcam	ab8216	AB_306361
CD68	KP1	Abcam	ab955	AB_307338
Procollagen-I	M-58	EMD Millipore	MAB1912	AB_94405
**mfIHC, xenograft mouse panel**				
GLI1	UMAB170	Origene	UM870063	AB_2629173
HLA-ABC	EMR8-5	Abcam	ab70328	AB_1269092
CD45	MEM-28	Abcam	ab8216	AB_306361
CD68	KP1	Abcam	ab955	AB_307338
Procollagen-I	M-58	EMD Millipore	MAB1912	AB_94405
**Immunostaining**				
GLI1	*Polyclonal*	Novus	NBP1-78259	AB_11030198
**Western immunoblotting**				
GLI1	1B9F8	US Biological	368745	AB_2889171
GLI2	*Polyclonal*	Invitrogen	PA5-79314	AB_2746430
MMP2	D8N9Y	Cell Signaling Technology	13132	AB_2798128
MMP9	D603H	Cell Signaling Technology	13667	AB_2798289
Procollagen-I	M-58	EMD Millipore	MAB1912	AB_94405
pSMAD2	138D4	Cell Signaling Technology	3108	AB_490941
pSMAD3	C25A9	Cell Signaling Technology	9520	AB_2193207
SMAD2/3	*Polyclonal*	Cell Signaling Technology	3102	AB_10698742
pSTAT3 (Tyr705)	*Polyclonal*	Cell Signaling Technology	9131	AB_331586
STAT3	79D7	Cell Signaling Technology	4904	AB_331269
β-actin	AC-15	Sigma-Aldrich	A5441	AB_476744
**Immunoprecipitation**				
STAT3	79D7	Cell Signaling Technology	4904	AB_331269
IgG	*Polyclonal*	Cell Signaling Technology	2729	AB_1031062

Abbreviations: RRID, Research Resource Identifier; mfIHC, multiplexed fluorescence immunohistochemistry.

Supplementary Table S4. Probes and siRNAs used in RNA-based assays

Target	Manufacturer	Catalog no. / Assay ID
**TaqMan probes**		
GLI1	Applied Biosystems	Hs00171790_m1
MMP2	Applied Biosystems	Hs01548727_m1
MMP9	Applied Biosystems	Hs00957562_m1
COL1A1	Applied Biosystems	Hs00164004_m1
STAT3	Applied Biosystems	Hs00374280_m1
PPIA	Applied Biosystems	Hs04194521_s1
**RNAscope probes**		
GLI1 (C1)	Advanced Cell Diagnostics	310991
MMP9 (C2)	Advanced Cell Diagnostics	311331-C2
MMP2 (C3)	Advanced Cell Diagnostics	311751-C3
COL1A1 (C4)	Advanced Cell Diagnostics	401891-C4
Positive control (4-plex)	Advanced Cell Diagnostics	321801
Negative control (dapB)	Advanced Cell Diagnostics	321831
**siRNAs**		
GLI1	Applied Biosystems	HSS178441
GLI2	Applied Biosystems	HSS178443
STAT3	Applied Biosystems	s743
Scrambled control (Ctrl)	Applied Biosystems	AM4611

Supplementary Table S5. Fluorophores used in multiplexed fluorescence assays

Fluorophore	Target	Manufacturer	Catalog no.
**mfIHC, human panel A**			
Opal 520	GLI1	Akoya Biosciences	FP1487001KT
Opal 540	CD105	Akoya Biosciences	FP1494001KT
Opal 570	Procollagen-I	Akoya Biosciences	FP1488001KT
Opal 620	CD68	Akoya Biosciences	FP1495001KT
Opal 650	CD90	Akoya Biosciences	FP1496001KT
Opal 690	CD45	Akoya Biosciences	FP1497001KT
**mfIHC, human panel B**			
Opal 480	pSTAT3	Akoya Biosciences	FP1500001KT
Opal 520	IL-6	Akoya Biosciences	FP1487001KT
Opal 570	Procollagen-I	Akoya Biosciences	FP1488001KT
Opal 620	CD68	Akoya Biosciences	FP1495001KT
Opal 690	CD45	Akoya Biosciences	FP1497001KT
**mfIHC, xenograft mouse panel**			
Opal 480	GLI1	Akoya Biosciences	FP1500001KT
Opal 520	CD68	Akoya Biosciences	FP1487001KT
Opal 570	CD45	Akoya Biosciences	FP1488001KT
Opal 690	Procollagen-I	Akoya Biosciences	FP1497001KT
Opal 780	HLA-ABC	Akoya Biosciences	FP1501001KT
**RNA *in situ* hybridization**			
Opal 520	GLI1 (C1)	Akoya Biosciences	FP1487001KT
Opal 570	MMP9 (C2)	Akoya Biosciences	FP1488001KT
Opal 620	MMP2 (C3)	Akoya Biosciences	FP1495001KT
Opal 690	COL1A1 (C4)	Akoya Biosciences	FP1497001KT

Abbreviations: mfIHC, multiplexed fluorescence immunohistochemistry.

Supplementary Table S6. Putative STAT3 binding sites within the GLI1 gene promoter

GAS-like element	Position* (bp)	Length (bp)	Sequence
1	-161 → -152	10	AACTCGAATT
2	-375 → -368	8	AAGCGATT
3	-567 → -558	10	TTCCCAAGAA
4	-639 → -630	10	TTAGTCCAAA
5	-673 → -665	9	TTACTAAAA
6	-702 → -686	17	TTACAGCAAGAAGAATT
7	-931 → -922	10	TTCTGTCTAA

Supplementary Table S7. GLI1 gene promoter fragments used in the chromatin immunoprecipitation (ChIP) assay

GAS-like element	Position* (bp)	Length (bp)	Primers
1	-295 → -94	202	Forward: 5'-TGTAGAGACGGGGTTTCAC-3'<br>Reverse: 5'-CAGAATCCTGGGCAGAACAT-3'
2	-526 → -360	167	Forward: 5'-CTCTGCCTCTCTGGGACATC-3'<br>Reverse: 5'-GGCAGAAGAATCGCTTGAAC-3'
3	-583 → -479	105	Forward: 5'-CAAAGCTCCCACCCAGTTC-3'<br>Reverse: 5'-CTGAGTGAGGGGAGGGGT-3'
4-6	-744 → -599	146	Forward: 5'-TCACACGAGTTCTCCCATCC-3'<br>Reverse: 5'-TGCCTGTGTGGTCTGTGTGT-3'
7	-1005 → -816	190	Forward: 5'-ACCAGCTTTCACCTTTGTGC-3'<br>Reverse: 5'-GGACAGCCCGTGTACAATCT-3'

Supplementary Table S8. GLI1 gene promoter fragments used in the luciferase assay

GAS-like element	Position* (bp)	Length (bp)	Primers
1, 2, 3, 4-6, 7	-952 → +32	984	Forward: 5'-TACCTCCTCCTCCACACTGTT-3'<br>Reverse: 5'-CTGATTGGTGGTGGGGTCAT-3'
1, 2, 3	-568 → +32	600	Forward: 5'-GTTCCCAAGAAGATCCCCAGAG-3'<br>Reverse: 5'-CTGATTGGTGGTGGGGTCAT-3'
1, 2	-524 → +32	556	Forward: 5'-CTGCCTCTCTGGGACATCATTT-3'<br>Reverse: 5'-CTGATTGGTGGTGGGGTCAT-3'
1	-161 → +32	193	Forward: 5'- AACTCGAATTCCGTGGCAGA-3'<br>Reverse: 5'-CTGATTGGTGGTGGGGTCAT-3'

GAS, gamma-interferon activated sequence.
*Position from the GLI1 gene start codon.

References

1. Arber DA, Orazi A, Hasserjian R, Thiele J, Borowitz MJ, Le Beau MM, et al. The 2016 revision to the World Health Organization classification of myeloid neoplasms and acute leukemia. Blood. 2016;127:2391-405.
2. Thiele J, Kvasnicka HM, Facchetti F, Franco V, van der Walt J, Orazi A. European consensus on grading bone marrow fibrosis and assessment of cellularity. Haematologica. 2005;90:1128-32.
3. Tefferi A, Guglielmelli P, Lasho TL, Gangat N, Ketterling RP, Pardanani A, et al. MIPSS70+version 2.0: mutation and karyotype-enhanced international prognostic scoring system for primary myelofibrosis. J Clin Oncol. 2018;36:1769-70.
4. Tefferi A, Nicolosi M, Mudireddy M, Lasho TL, Gangat N, Begna KH, et al. Revised cytogenetic risk stratification in primary myelofibrosis: analysis based on 1002 informative patients. Leukemia. 2018;32:1189-99.
